# The impact of the SARS-CoV-2 pandemic on cause-specific mortality patterns: a systematic literature review

**DOI:** 10.1007/s10389-022-01755-7

**Published:** 2022-09-26

**Authors:** Francesco Sanmarchi, Francesco Esposito, Emanuele Adorno, Francesco De Dominicis, Maria Pia Fantini, Davide Golinelli

**Affiliations:** Department of Biomedical and Neuromotor Sciences (DIBINEM), Alma Mater Studiorum—Università di Bologna, Via San Giacomo 12, 40126 Bologna, Italy

**Keywords:** COVID-19, Mortality, Systematic review

## Abstract

**Background:**

Understanding the effects of the COVID-19 pandemic on cause-specific mortality should be a priority, as this metric allows for a detailed analysis of the true burden of the pandemic. The aim of this systematic literature review is to estimate the impact of the pandemic on different causes of death, providing a quantitative and qualitative analysis of the phenomenon.

**Methods:**

We searched MEDLINE, Scopus, and ProQuest for studies that reported cause-specific mortality during the COVID-19 pandemic, extracting relevant data.

**Results:**

A total of 2413 articles were retrieved, and after screening 22 were selected for data extraction. Cause-specific mortality results were reported using different units of measurement. The most frequently analyzed cause of death was cardiovascular diseases (*n* = 16), followed by cancer (*n* = 14) and diabetes (*n* = 11). We reported heterogeneous patterns of cause-specific mortality, except for suicide and road accident.

**Conclusions:**

Evidence on non-COVID-19 cause-specific deaths is not exhaustive. Reliable scientific evidence is needed by policymakers to make the best decisions in an unprecedented and extremely uncertain historical period. We advocate for the urgent need to find an international consensus to define reliable methodological approaches to establish the true burden of the COVID-19 pandemic on non-COVID-19 mortality.

**Supplementary Information:**

The online version contains supplementary material available at 10.1007/s10389-022-01755-7.

## Introduction

During the SARS-CoV-2 pandemic, a surge in overall deaths has been recorded in many countries, most of them probably attributable to COVID-19 (Wang et al. 2022). Numerous researchers investigated the patterns of COVID-19-related excess mortality at the international, national, and subnational levels (Kontis et al. 2020; Ahmad and Anderson 2021), with most of the studies focusing on all-cause mortality. Excess mortality analysis investigates the difference between the reported number of deaths within a given time in a location, and the number of expected deaths in “normal conditions” (often estimated using the same period in the preceding year or averaged over several preceding years) (Beaney et al. 2020). All-cause mortality is considered a strong and comprehensive indicator that reflects the full burden of the pandemic as it considers the pandemic’s direct and indirect effects on individuals, healthcare systems, and society as a whole (Beaney et al. 2020) and allows to nullify the differences in COVID-19 reporting and testing strategies and in the misclassification of the cause of death on certificates (Sanmarchi et al. 2021).

Although this information is relevant, understanding the effects of the pandemic on cause-specific mortality is crucial and should be deeply assessed, as this metric allows for a more detailed analysis of the true impact of the pandemic on people's health. Specifically, through cause-specific mortality, researchers analyze the pandemic indirect effects on other communicable and non-communicable diseases. Thus, investigating cause-specific mortality would lead to overcoming all-cause excess mortality limitations and better understanding the true impact of the pandemic on society (Beaney et al. 2020).

On the one hand, the spreading of the SARS-CoV-2 infection has directly caused a huge number of deaths, especially in the older population (Banerjee et al. 2020; Gibertoni et al. 2021), becoming the third leading cause of death in 2020 (Ahmad and Anderson 2021); on the other hand, it may have indirectly changed the burden of mortality from other causes. For instance, the adoption of non-pharmaceutical interventions (e.g., physical-distancing, mask mandates, school closings) to control the COVID-19 pandemic determined a decreased risk of death from certain other causes (e.g., car accidents or work-related accidents) (Yasin et al. 2021). Conversely, overwhelmed healthcare systems and changes to clinical pathways may have affected standard care, thus increasing the number of deaths for many acute and chronic diseases (Santi et al. 2021; Golinelli et al. 2021) and possibly leading to an increase in mortality from underdiagnosed diseases (Arolas et al. 2021; Aron and Muellbauer 2022).

To the best of our knowledge, there are no systematic literature reviews that have analyzed trends of cause-specific mortality during the SARS-CoV-2 pandemic. Such evidence should guide policymakers and the healthcare systems and help them dealing with the pandemic and its aftermath. Therefore, we undertook a systematic literature review to summarize current knowledge on cause-specific mortality during the pandemic and we aimed at estimating the impact of this global health crisis on different causes of death, providing a quantitative and qualitative analysis of the phenomenon. Specifically, we evaluated the number of papers published for each cause of death, evaluated whether the effects of the pandemic are consistent among different countries, provided insights into the models used to estimate the expected mortality, and discussed the cause-specific mortality estimates.

## Methods

We developed a search strategy, following the Preferred Reporting Items for Systematic Reviews (PRISMA) approach (Page et al. 2020), to identify studies that reported cause-specific mortality during the COVID-19 pandemic.

We searched the electronic databases MEDLINE , Scopus, and ProQuest (from January 1, 2020, to July 31, 2022).

We used the following search string for MEDLINE:

*2020/01/01:2022/07/31[Date - Publication] AND (("excess"[All Fields] OR "excesses"[All Fields] OR "excessive"[All Fields] OR "excessively"[All Fields]) AND (("mortality"[MeSH Terms] OR "mortality"[All Fields] OR "mortalities"[All Fields] OR "mortality"[MeSH Subheading]) AND ("sars cov 2"[MeSH Terms] OR "sars cov 2"[All Fields] OR "covid"[All Fields] OR "covid 19"[MeSH Terms] OR "covid 19"[All Fields]) AND ("causative"[All Fields] OR "causatively"[All Fields] OR "causatives"[All Fields] OR "cause"[All Fields] OR "caused"[All Fields] OR "causing"[All Fields] OR "etiology"[MeSH Subheading] OR "etiology"[All Fields] OR "causes"[All Fields] OR "causality"[MeSH Terms] OR "causality"[All Fields] OR "disease-specific"[All Fields]))).*

We used the following search string for Scopus:

( excess* ) AND ( ( mortality ) OR ( deaths ) ) AND ( covid-19 ) AND ( cause AND specific )

We used the following search terms for ProQuest:

*“(excess mortality) AND (COVID-19) AND (cause-specific mortality)” *, limiting the search to peer-reviewed articles in academic journals.

### Inclusion and exclusion criteria

We adopted the following inclusion criteria: (i) original article, (ii) assessment of at least one cause-specific mortality during the pandemic period, (iii) assessed causes of deaths using the 10th revision of the International Classification of Diseases (ICD-10), (iv) reporting of at least one of the following outcomes: cause-specific mortality estimates or cause-specific excess mortality, and (v) full-length articles. We excluded articles based on the following criteria: (i) preprints of articles, (ii) reviews, and (iii) non-English-language articles.

### Data extraction and analysis

Two independent investigators (EA, FE) evaluated each record to determine eligibility. All disagreements were discussed with an independent arbiter (FS) to reach consensus. The investigators (EA, FE) independently extracted the data. Disagreements were discussed with FS. Relevant data extracted from each article were: publication year, data stratification, territory, country income level (World Bank Index (Fantom and Serajuddin 2016)), results standardization, results’ level of aggregation, all-cause excess mortality, and cause-specific mortality (values and unit of measure).

When extracting cause-specific mortality data, we reported confidence intervals if available, if not, we reported the percentage variation and the respective *p*-value and if the *p*-value was not included, we reported the estimates. For the sake of completeness, we reported all the results (Tables 1, 2, 3, and 4 and supplemental material), but we limited the discussion only to those studies that reported statistical significance.

### Quality assessment

The evaluation of the included studies was performed using the Newcastle–Ottawa Quality Assessment Scale (NOS) developed by Wells and colleagues (Wells et al. 2022). NOS contains eight items within three domains and the total maximum score is 9. A study with a score from 7–9 has fair to good quality, 4–6 high risk, and 0–3 very high risk of bias.

## Results

A total of 705 were retrieved from MEDLINE, 2380 from Scopus and 379 articles were retrieved from ProQuest. After merging and duplicates removal, 2413 articles were screened. Out of those articles, 1896 were ruled out after title screening. Of the remaining 517 articles, 439 were removed after abstract screening.

**Fig. 1:**
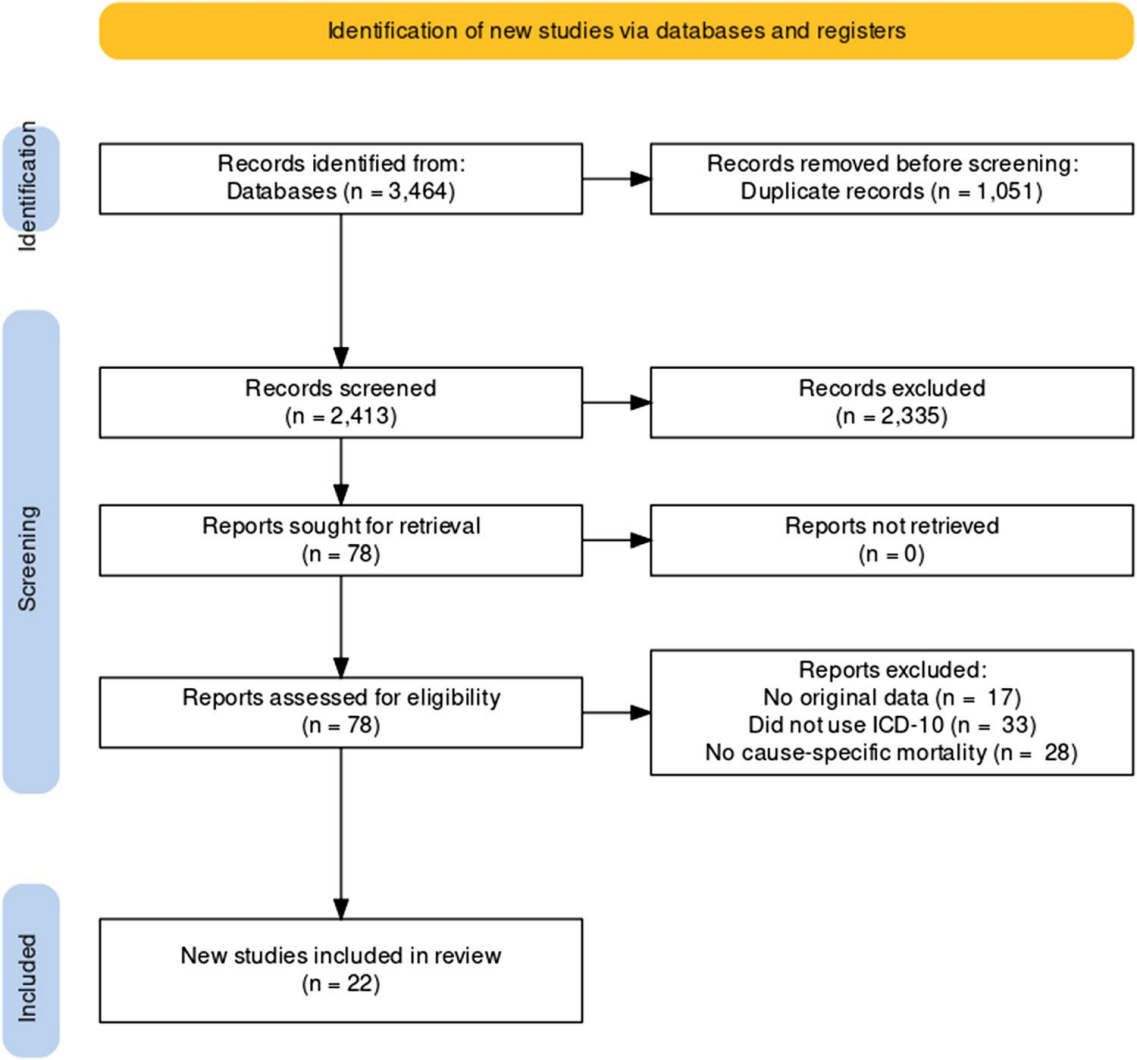
PRISMA flowchart

A total of 78 articles were included in the full-text review process (Fig. 1). Of these, 56 were excluded after the full-text screening. We extracted relevant data from the final set of 22 articles (Brant et al. 2020; Fernandes et al. 2021; Décarie and Michaud 2021; Liu et al. 2021; Li et al. 2021; Shoaib et al. 2021; Wu et al. 2021a, b; Al Wahaibi et al. 2021; Wang et al. 2021; Wu et al. 2021a, b; Kontopantelis et al. 2021; Sharma et al. 2021; Faust et al. 2021; Grande et al. 2022; Palacio-Mejía et al. 2022; Gobiņa et al. 2022; Perotti et al. 2022; Jardim et al. 2022; Glei 2022; Orellana and de Souza 2022; Odd et al. 2022; Chen et al. 2022).

Among the 22 articles selected for data extraction, one was published in 2020 (Brant et al. 2020), 12 were published in 2021 (Fernandes et al. 2021; Décarie and Michaud 2021; Liu et al. 2021; Li et al. 2021; Shoaib et al. 2021; Wu et al. 2021a, b; Al Wahaibi et al. 2021; Wang et al. 2021; Wu et al. 2021a, b; Kontopantelis et al. 2021; Sharma et al. 2021; Faust et al. 2021) and nine were published in 2022 (Grande et al. 2022; Palacio-Mejía et al. 2022; Gobiņa et al. 2022; Perotti et al. 2022; Jardim et al. 2022; Glei 2022; Orellana and de Souza 2022; Odd et al. 2022; Chen et al. 2022). All the included studies used data from official governmental sources to perform their analysis. Studies’ characteristics are reported in Table S1.

Out of the nine countries investigated by the included studies, two of them (Brazil and Mexico) are classified as upper-middle-income by the World Bank classification system, while the others are classified as high-income (Canada, China, Italy, Latvia, Oman, United Kingdom, and United States of America). The studies included in our review have study settings with heterogeneous granularity. For instance, studies investigate mortality at a city level (Brant et al. 2020; Fernandes et al. 2021; Liu et al. 2021; Li et al. 2021; Perotti et al. 2022), subnational region/state level (Décarie and Michaud 2021; Liu et al. 2021; Shoaib et al. 2021; Kontopantelis et al. 2021; Sharma et al. 2021; Chen et al. 2022) and country levels (Shoaib et al. 2021; Wu et al. 2021a, b; Al Wahaibi et al. 2021; Wang et al. 2021; Wu et al. 2021a, b; Kontopantelis et al. 2021; Faust et al. 2021; Grande et al. 2022; Palacio-Mejía et al. 2022; Gobiņa et al. 2022; Jardim et al. 2022; Glei 2022; Orellana and de Souza 2022; Odd et al. 2022).

All but three studies analyzed timeframes within the year 2020. All the included studies reported the extensions of the historical data used to estimate the expected deaths. The mean length of the study period was 226.3 ± 149.5 days. The mean length of the historical data used to obtain the expected number of deaths was 5 ± 2 years.

All studies reported the methods used to estimate the expected mortality for the respective study period. The Poisson regression model (*n* = 7) (Brant et al. 2020; Décarie and Michaud 2021; Li et al. 2021; Shoaib et al. 2021; Sharma et al. 2021; Jardim et al. 2022; Palacio-Mejía et al. 2022) was the most widely employed method, followed by the mean number of deaths of the previous years (*n* = 4) (Shoaib et al. 2021; Wang et al. 2021; Grande et al. 2022; Perotti et al. 2022), the Farrington surveillance algorithm (*n* = 3) (Liu et al. 2021; Al Wahaibi et al. 2021: Wu et al. 2021a, b), and the negative binomial regression model (*n* = 3) (Kontopantelis et al. 2021; Glei 2022; Odd et al. 2022). Other methods included the autoregressive integrated moving average (ARIMA) model (*n* = 2) (Faust et al. 2021; Chen et al. 2022) and generalized additive modeling (GAM) (*n* = 2; Gobina et al. 2022; Orellana and de Souza 2022). One study (Fernandes et al. 2021) reported using the 2019 number of deaths as the expected number of deaths of 2020.

Of all included studies, 13 (Fernandes et al. 2021; Li et al. 2021; Shoaib et al. 2021; Wu et al. 2021a, b; Al Wahaibi et al. 2021; Wang et al. 2021; Wu et al. 2021a, b; Kontopantelis et al. 2021; Grande et al. 2022; Perotti et al. 2022; Jardim et al. 2022; Glei 2022; Orellana and de Souza 2022) studies reported stratified results by socio-demographic variables. Specifically, ten studies stratified by sex (Fernandes et al. 2021; Wu et al. 2021a, b; Al Wahaibi et al. 2021; Wang et al. 2021; Wu et al. 2021a, b; Kontopantelis et al. 2021; Grande et al. 2022; Perotti et al. 2022; Glei 2022; Orellana and de Souza 2022), ten by age (Fernandes et al. 2021; Wu et al. 2021a, b; Al Wahaibi et al. 2021; Wang et al. 2021; Wu et al. 2021a, b; Kontopantelis et al. 2021; Grande et al. 2022; Perotti et al. 2022; Glei 2022; Orellana and de Souza 2022), two by month (Wang et al. 2021; Faust et al. 2021), two by healthcare setting (Shoaib et al. 2021; Al Wahaibi et al. 2021), one by type of occupation(Li et al. 2021) and one by quintile of deprivation (Kontopantelis et al. 2021). Only six studies (Fernandes et al. 2021; Wang et al. 2021; Grande et al. 2022; Perotti et al. 2022; Glei 2022; Jardim et al. 2022) reported standardized mortality rates.

Cause-specific mortality results were reported using different units of measurement. Specifically, three studies (Brant et al. 2020; Li et al. 2021; Grande et al. 2022) analyzed the percentage change in mortality with respect to the comparison period, seven studies (Décarie and Michaud 2021; Kontopantelis et al. 2021; Sharma et al. 2021; Palacio-Mejía et al. 2022; Gobiņa et al. 2022; Glei 2022; Chen et al. 2022) analyzed the absolute number of excess deaths, two studies (Fernandes et al. 2021; Al Wahaibi et al. 2021) reported the standardized mortality ratio, two studies (Wu et al. 2021a, b) reported both the total number of excess deaths and percentage variation, six studies (Liu et al. 2021; Al Wahaibi et al. 2021; Faust et al. 2021; Perotti et al. 2022; Jardim et al. 2022; Odd et al. 2022) reported the observed-to-expected ratio, and one study (Shoaib et al. 2021) reported the incidence rate ratio.

Of the 22 included studies, 15 report significance and/or confidence intervals (Brant et al. 2020; Fernandes et al. 2021; Liu et al. 2021; Li et al. 2021; Shoaib et al. 2021; Al Wahaibi et al. 2021; Wang et al. 2021; Kontopantelis et al. 2021; Faust et al. 2021; Palacio-Mejía et al. 2022; Gobiņa et al. 2022; Perotti et al. 2022; Jardim et al. 2022; Orellana and de Souza 2022; Odd et al. 2022). Moreover, there was no consensus among the included studies on the ICD-10 codes used to group causes of death.

### Cause-specific mortality

Table 1 reports the number of studies which analyzed a specific cause of death, and for each specific cause shows the number of studies which included the statistical significance levels with their results (e.g., 11 out of 16 studies on cardiovascular-specific mortality reported 95% confidence intervals in their estimates).

**Table 1:** Number of studies that investigated a specific cause of death

Cause of death	Studies investigating the specific cause of death	Studies reporting statistical significance
Cardiovascular diseases	16	11
Cancer	14	10
Diabetes mellitus	11	7
Suicides	9	6
Cerebrovascular diseases	9	4
Road accidents	7	5
Chronic lower respiratory diseases	6	5
Diseases of the respiratory system (excluding COVID-19)	5	4
Infectious diseases (excluding COVID-19)	5	4
Ischemic heart disease	4	3
Unintentional injuries	4	2
Influenza and pneumonia	4	2
Alzheimer’s disease	4	1
Digestive system disease	3	3
Hypertensive diseases	3	2
Kidney diseases	3	2
Dementia and Alzheimer’s disease	3	1
Mental and behavioral disorders	2	2
Diseases of the nervous system and sense organs	2	2
Diseases of the genitourinary system	2	2

The most frequently analyzed causes of deaths were cardiovascular diseases (*n* = 16, Table 2) (Brant et al. 2020; Fernandes et al. 2021; Décarie and Michaud 2021; Liu et al. 2021; Li et al. 2021; Shoaib et al. 2021; Wu et al. 2021a, b; Al Wahaibi et al. 2021; Wang et al. 2021; Wu et al. 2021a, b; Sharma et al. 2021; Gobiņa et al. 2022; Perotti et al. 2022; Jardim et al. 2022; Glei 2022; Chen et al. 2022), followed by cancer (*n* = 14, Table 3) (Fernandes et al. 2021; Décarie and Michaud 2021; Liu et al. 2021; Li et al. 2021; Wu et al. 2021a, b; Kontopantelis et al. 2021; Grande et al. 2022; Palacio-Mejía et al. 2022; Gobiņa et al. 2022; Perotti et al. 2022; Jardim et al. 2022; Glei 2022; Odd et al. 2022; Chen et al. 2022), diabetes (*n* = 11, Table 4) (Fernandes et al. 2021; Décarie and Michaud 2021; Liu et al. 2021; Li et al. 2021; Wu et al. 2021a, b; Grande et al. 2022; Palacio-Mejía et al. 2022; Gobiņa et al. 2022; Perotti et al. 2022; Glei 2022; Chen et al. 2022), and suicide (*n* = 9) (Décarie and Michaud 2021; Liu et al. 2021; Shoaib et al. 2021; Faust et al. 2021; Grande et al. 2022; Palacio-Mejía et al. 2022; Perotti et al. 2022; Glei 2022; Odd et al. 2022). Cerebrovascular and road accident deaths were also frequently investigated (Table 1).

**Table 2:** Excess deaths from cardiovascular diseases

Study title	Territory	Level of aggregation	Specific aggregation	Expected vs observed	Unit of measure
Excess of cardiovascular deaths during the COVID-19 pandemic in Brazilian capital cities	Country, Brazil	City	São Paulo	10.1% (5.2, 15.3)	% variation (95% CI)
		City	Rio de Janeiro	−7.1% (−11.9, −1.9)	% variation (95% CI)
		City	Fortaleza	12.6% (2.4, 23.8)	% variation (95% CI)
		City	Recife	6.6% (−4.4, 18.8)	% variation (95% CI)
		City	Belém	43.6% (27.3, 62)	% variation (95% CI)
		City	Manaus	46.1% (29.5, 64.9)	% variation (95% CI)
Excess mortality by specific causes of deaths in the city of São Paulo, Brazil, during the COVID-19 pandemic	City, San-Paolo, Brazil	Gender	Males	0.9 (0.75, 1.13)	Standardized mortality ratio (95% CI)
		Gender	Females	0.9 (0.68, 1.15)	Standardized mortality ratio (95% CI)
Counting the dead: COVID-19 and mortality in Quebec and British Columbia during the first wave	Territories, Canada	Territory	British Columbia	−67.1	Excess deaths
		Territory	Quebec	−279.0	Excess deaths
Excess mortality in Wuhan city and other parts of China during the 3 months of the COVID-19 outbreak: findings from nationwide mortality registries	Territories, China	City	Wuhan	1.29 (1.05, 1.65)	Rate ratio (95% CI)
		Territory	Hubei without Wuhan	0.98 (0.83, 1.18)	Rate ratio (95% CI)
		Territory	China without Hubei	0.95 (0.86, 1.07)	Rate ratio (95% CI)
Temporal dynamic in the impact of COVID- 19 outbreak on cause-specific mortality in Guangzhou, China	City, China	Aggregated		1.9 (−1.5, 5.0)	% variation (95% CI)
		Gender	Males	1.6 (−1.9, 4.6)	% variation (95% CI)
		Gender	Females	2.3 (−0.8, 5.4)	% variation (95% CI)
		Age group	< 25	−24.4 (−46.5, −5.3)	% variation (95% CI)
		Age group	25–44	12.9 (4.6, 20.5)	% variation (95% CI)
		Age group	45−64	-9.6 (-13.5, -6.0)	% variation (95% CI)
		Age group	65−74	5.3 (1.6, 9.0)	% variation (95% CI)
		Age group	75−84	−3.5 (−7.0, −0.2)	% variation (95% CI)
		Age group	85+	11.1 (7.8, 14.2)	% variation (95% CI)
		Setting of care	Hospital	−3.8 (−7.4, −0.5)	% variation (95% CI)
		Setting of care	Outside hospitals	3.6 (0.4, 6.9)	% variation (95% CI)
		Marital status	Unmarried	13.5 (7.1, 18.9)	% variation (95% CI)
		Marital status	Married	−1.7 (−5.1, 1.5)	% variation (95% CI)
		Marital status	Divorced	4.2 (−3.2, 10.7)	% variation (95% CI)
		Marital status	Widowed	4.0 (0.3, 7.2)	% variation (95% CI)
		Occupation class	Gold-collar	−15.5 (−25.9, −7.1)	% variation (95% CI)
		Occupation class	White-collar	−10.3 (−18.5, −3.0)	% variation (95% CI)
		Occupation class	Pink-collar	3.8 (−4.5, 10.7)	% variation (95% CI)
		Occupation class	Blue-collar	−3.1 (−6.5, −0.2)	% variation (95% CI)
		Occupation class	Others	3.8 (0.7, 6.8)	% variation (95% CI)
Substantial decline in hospital admissions for heart failure accompanied by increased community mortality during COVID-19 pandemic	Country, England	Setting of care	Hospital	0.71 (−0.08, −1.23)	Incidence rate ratio (95% CI)
		Setting of care	Home	1.31 (1.24, 1.39)	Incidence rate ratio (95% CI)
		Setting of care	Care homes and hospices	1.28 (1.18, 1.40)	Incidence rate ratio (95% CI)
Place and underlying cause of death during the covid-19 pandemic: retrospective cohort study of 3.5 million deaths in England and Wales, 2014 to 2020	Countries, England and Wales	Aggregated		2225 (+9%)	Excess deaths (% variation)
		Setting of care	Home	2485 (+26%)	Excess deaths (% variation)
		Setting of care	Care home or hospice	1211 (+31%)	Excess deaths (% variation)
		Setting of care	Hospital	−1398 (−13%)	Excess deaths (% variation)
Effects of COVID-19 on mortality: a 5-year population-based study in Oman	Country, Oman	Aggregated		0	Observed-to-expected ratio (95% CI)
		Setting of care	Home	0	Observed-to-expected ratio (95% CI)
		Setting of care	Hospital	0	Observed-to-expected ratio (95% CI)
Cardiovascular-related deaths at the beginning of the COVID-19 outbreak: a prospective analysis based on the UK Biobank	Country, Oman	Time period	March	1.19 (1.00, 1.40)	Standardized mortality ratio (95% CI)
		Time period	April	0.98 (0.81, 1.17)	Standardized mortality ratio (95% CI)
		Time period	May	0.67 (0.54, 0.83)	Standardized mortality ratio (95% CI)
		Time period	June	0.87 (0.70, 1.06)	Standardized mortality ratio (95% CI)
Place and causes of acute cardiovascular mortality during the COVID-19 pandemic	Countries, England and Wales	Aggregated		2085 (+8%)	Excess deaths (% variation)
		Gender	Males	1182 (+8%)	Excess deaths (% variation)
		Gender	Females	948 (+7%)	Excess deaths (% variation)
		Age	18–49	176 (+17%)	Excess deaths (% variation)
		Age	50–59	248 (+14%)	Excess deaths (% variation)
		Age	60–69	468 (+15%)	Excess deaths (% variation)
		Age	70–79	688 (+11%)	Excess deaths (% variation)
		Age	80+	734 (+5%)	Excess deaths (% variation)
		Setting of care	Home	2279 (+35%)	Excess deaths (% variation)
		Setting of care	Care home and hospice	1095 (+32%)	Excess deaths (% variation)
		Setting of care	Hospital	50 (0%)	Excess deaths (% variation)
Excess cerebrovascular mortality in the United States during the COVID-19 pandemic	States, USA	Aggregated		6367	Excess deaths
Excess mortality associated with the COVID-19 pandemic in Latvia: a population-level analysis of all-cause and noncommunicable disease deaths in 2020	Country, Latvia	Aggregated		1309 (88, 2476)	Excess deaths (% variation)
Impact of the COVID-19 pandemic on total and cause-specific mortality in Pavia, Northern Italy	Province, Italy	Sex	Males	0.89 (0.83, 0.96)	Observed-to-expected ratio (95% CI)
		Sex	Females	0.96 (0.90, 1.01)	Observed-to-expected ratio (95%CI)
		Sex and age	M 50–64	0.93 (0.72, 10.2)	Observed-to-expected ratio (95% CI)
		Sex and age	M 65–79	1.00 (0.84, 1.19)	Observed-to-expected ratio (95% CI)
		Sex and age	M 80+	0.83 (0.76, 0.91)	Observed-to-expected ratio (95% CI)
		Sex and age	F 50–64	1.06 (0.69, 1.63)	Observed-to-expected ratio (95% CI)
		Sex and age	F 65–79	1.01 (0.83, 1.22)	Observed-to-expected ratio (95% CI)
		Sex and age	F 80+	0.95 (0.89, 1.00)	Observed-to-expected ratio (95% CI)
COVID-19 in Brazil in 2020: impact on deaths from cancer and cardiovascular diseases	Country, Brazil	Aggregated		0.90 (0.90, 0.91)	Observed-to-expected ratio (95% CI)
		Territory	North	0.91 (0.89, 0.93)	Observed-to-expected ratio (95% CI)
		Territory	Northeast	0.90 (0.89, 0.91)	Observed-to-expected ratio (95% CI)
		Territory	Southeast	0.90 (0.90, 0.91)	Observed-to-expected ratio (95% CI)
		Territory	South	0.92 (0.91, 0.93)	Observed-to-expected ratio (95% CI)
		Territory	Midwest	0.89 (0.87, 0.91)	Observed-to-expected ratio (95% CI)
The US midlife mortality crisis continues: excess cause-specific mortality during 2020	Country, USA	Sex	Males	15,943	Excess deaths
		Sex	Females	10,566	Excess deaths
		Sex and age	M < 15	-	Excess deaths
		Sex and age	M 15–24	-	Excess deaths
		Sex and age	M 25–44	1589	Excess deaths
		Sex and age	M 45–64	7122	Excess deaths
		Sex and age	M 65–74	2534	Excess deaths
		Sex and age	M ≥ 75	4698	Excess deaths
		Sex and age	F < 15	-	Excess deaths
		Sex and age	F 15–24	21	Excess deaths
		Sex and age	F 25–44	607	Excess deaths
		Sex and age	F 45–64	2110	Excess deaths
		Sex and age	F 65–74	2294	Excess deaths
		Sex and age	F ≥75	5535	Excess deaths
Excess natural-cause deaths in California by cause and setting: March 2020 through February 2021	State, USA	Care setting	In hospital	−1930 (−2520, −1336)	Excess deaths (95% CI)
		Care setting	Out of hospital	7649 (5762, 9539)	Excess deaths (95% CI)

As for cardiovascular mortality (Table 2), the included studies reported heterogeneous patterns of cause-specific mortality in terms of data stratification and trends. For instance, the study by Brant et al. (2020) investigated cardiovascular mortality in six major Brazilian cities and reported changes in mortality ranging from +46.1% (29.5–64.9, 95% CI) in Manaus to −7.1% (−11.9 to−1.9, 95% CI) in Rio de Janeiro. The study by Li et al. (2021) investigated patterns of cardiovascular mortality for different age groups and working/occupation class, and reported changes in mortality ranging from +12.9% (4.6–20.5, 95% CI) in the age class 25–44 years, to −24.4% (−46.5 to 5.3, 95% CI) in the age class < 25 years.

As for cancer mortality (Table 3), the included studies reported heterogenous patterns of cause-specific mortality. Décarie and Michaud (2021) reported a decrease in excess deaths both in British Columbia and in Quebec regions (−45.0 and −570.5 excess deaths respectively), while Liu et al. (2021) reported no significant changes in cancer mortality in Wuhan city, Hubei Region without Wuhan, and China without Hubei region (1.02, 1.08 and 0.99 mortality rate ratios respectively).

**Table 3:** Excess deaths from cancers

Study title	Territory	Level of aggregation	Specific aggregation	Expected vs observed	Unit of measure
Excess mortality by specific causes of deaths in the city of São Paulo, Brazil, during the COVID-19 pandemic	City, San-Paolo, Brazil	Gender	Males	0.9 (0.66, 1.09)	Standardized mortality ratio (95%CI)
		Gender	Females	0.9 (0.67, 1.20)	Standardized mortality ratio (95%CI)
Counting the dead: COVID-19 and mortality in Quebec and British Columbia during the first wave	Territories, Canada	Territory	British Columbia	−45.0	Excess deaths
		Territory	Quebec	−570.5	Excess deaths
Excess mortality in Wuhan city and other parts of China during the three months of the COVID-19 outbreak: findings from nationwide mortality registries	Territories, China	City	Wuhan	1.02 (0.81, 1.33)	Rate ratio (95% CI)
		Territory	Hubei without Wuhan	1.08 (0.94, 1.25)	Rate ratio (95% CI)
		Territory	China without Hubei	0.99 (0.93, 1.06)	Rate ratio (95% CI)
Temporal dynamic in the impact of COVID- 19 outbreak on cause-specific mortality in Guangzhou, China	City, China	Aggregated		1.3 (−2.8, 4.8)	% variation (95% CI)
		Gender	Males	1.4 (−2.4, 5.1)	% variation (95% CI)
		Gender	Females	1.0 (−3.0, 4.6)	% variation (95% CI)
		Age group	< 25	−13.6 (−29.4, −0.4)	% variation (95% CI)
		Age group	25–44	1.7 (−4.5, 7.5)	% variation (95% CI)
		Age group	45–64	0.6 (−3.7, 4.6)	% variation (95% CI)
		Age group	65–74	8.6 (4.2, 12.7)	% variation (95% CI)
		Age group	75–84	−4.7 (−9.1, −0.5)	% variation (95% CI)
		Age group	85+	0.2 (−4.9, 5.2)	% variation (95% CI)
		Setting of care	Hospital	−5.4 (−9.5, −1.8)	% variation (95% CI)
		Setting of care	Outside hospitals	7.6 (3.6, 11.2)	% variation (95% CI)
		Marital status	Unmarried	−1.3 (−8.9, 5.3)	% variation (95% CI)
		Marital status	Married	−0.2 (−4.0, 3.4)	% variation (95% CI)
		Marital status	Divorced	10.8 (2.8, 17.7)	% variation (95% CI)
		Marital status	Widowed	−0.1 (−4.7, 4.6)	% variation (95% CI)
		Occupation class	Gold-collar	−19.0 (−27.8, −11.1)	% variation (95% CI)
		Occupation class	White-collar	−8.1 (−14.9, −1.9)	% variation (95% CI)
		Occupation class	Pink-collar	−12.2 (−19.2, −5.6)	% variation (95% CI)
		Occupation class	Blue-collar	3.8 (−0.8, 7.7)	% variation (95% CI)
		Occupation class	Others	0.3 (−3.8, 3.9)	% variation (95% CI)
Place and underlying cause of death during the COVID-19 pandemic: retrospective cohort study of 3.5 million deaths in England and Wales, 2014 to 2020	Countries, England and Wales	Aggregated		687 (+1%)	Excess deaths (% variation)
		Setting of care	Home	5963 (+40%)	Excess deaths (% variation)
		Setting of care	Care home or hospice	−1495 (−10%)	Excess deaths (% variation)
		Setting of care	Hospital	−4088 (−24%)	Excess deaths (% variation)
Excess deaths from COVID-19 and other causes by region, neighbourhood deprivation level and place of death during the first 30 weeks of the pandemic in England and Wales: A retrospective registry study	Countries, England and Wales	Aggregated		1668 (289, 3047)	Excess deaths (95% CI)
		Gender	Males	857 (531, 1183)	Excess deaths (95% CI)
		Gender	Females	812 (527, 1097)	Excess deaths (95% CI)
		Age group	0–4	−5 (−9, −1)	Excess deaths (95% CI)
		Age group	15–44	20 (6, 34)	Excess deaths (95% CI)
		Age group	45–65	417 (329, 505)	Excess deaths (95% CI)
		Age group	65–74	486 (358, 615)	Excess deaths (95% CI)
		Age group	75–84	681 (517, 845)	Excess deaths (95% CI)
		Age group	85+	−500 (−621, −378)	Excess deaths (95% CI)
		Territory	North east	67 (39, 94)	Excess deaths (95% CI)
		Territory	North west	168 (107, 228)	Excess deaths (95% CI)
		Territory	Yorkshire & Humber	128 (79, 176)	Excess deaths (95% CI)
		Territory	East Midlands	203 (160, 247)	Excess deaths (95% CI)
		Territory	West Midlands	293 (242, 344)	Excess deaths (95% CI)
		Territory	East of England	−39 (−92, 14)	Excess deaths (95% CI)
		Territory	London	116 (71, 162)	Excess deaths (95% CI)
		Territory	South East Coast	359 (316, 402)	Excess deaths (95% CI)
		Territory	South Central	147 (110, 183)	Excess deaths (95% CI)
		Territory	South West	327 (274, 380)	Excess deaths (95% CI)
		Territory	Wales	−30 (−62, 2)	Excess deaths (95% CI)
		Deprivation quintiles	1 (least deprived)	453 (357, 549)	Excess deaths (95% CI)
		Deprivation quintiles	2	−71 (31, 172)	Excess deaths (95% CI)
		Deprivation quintiles	3	228 (128, 328)	Excess deaths (95% CI)
		Deprivation quintiles	4	446 (353, 538)	Excess deaths (95% CI)
		Deprivation quintiles	5 (most deprived)	542 (448, 635)	Excess deaths (95% CI)
		Setting of care	Care home	−917 (−1000, −835)	Excess deaths (95% CI)
		Setting of care	Home	10665 (10498, 10833)	Excess deaths (95% CI)
		Setting of care	Hospice	−2186 (−2278, −2094)	Excess deaths (95% CI)
		Setting of care	Hospital	−6655 (−6854, −6456)	Excess deaths (95% CI)
		Setting of care	Other/ unknown	749 (735, 762)	Excess deaths (95% CI)
Variation in cause-specific mortality rates in Italy during the first wave of the COVID-19 pandemic: a study based on nationwide data	Country, Italy	Aggregated		−4.3%	% variation
		Gender	Males	−5.7%	% variation
		Gender	Females	−2.9%	% variation
Leading causes of excess mortality in Mexico during the COVID-19 pandemic 2020–2021: a death certificates study in a middle-income country	Country, Mexico	Aggregated		−10,126 (−19,044, −1209)	Excess deaths (95% CI)
Excess mortality associated with the COVID-19 pandemic in Latvia: a population-level analysis of all-cause and noncommunicable disease deaths in 2020	Country, Latvia	Aggregated		208 (−656, 1025)	Excess deaths (95% CI)
Impact of the COVID-19 pandemic on total and cause-specific mortality in Pavia, Northern Italy	Province, Italy	Sex	Males	0.86 (0.80, 0.92)	Standardized mortality ratio (95%CI)
		Sex	Females	0.97 (0.90, 1.04)	Standardized mortality ratio (95% CI)
		Sex and ageTO HERE	M 50-64	0.77 (0.65, 0.92)	Standardized mortality ratio (95% CI)
		Sex and age	M 65-79	0.85 (0.77, 0.94)	Standardized mortality ratio (95% CI)
		Sex and age	M 80+	0.93 (0.84, 1.04)	Standardized mortality ratio (95% CI)
		Sex and age	F 50-64	0.86 (0.71, 1.05)	Standardized mortality ratio (95% CI)
		Sex and age	F 65-79	0.98 (0.86, 1.11)	Standardized mortality ratio (95% CI)
		Sex and age	F 80+	0.97 (0.88, 1.08)	Standardized mortality ratio (95% CI)
Covid-19 in Brazil in 2020: impact on deaths from cancer and cardiovascular diseases	Country, Brazil	Aggregated		0.90 (0.90, 0.91)	Observed-to-expected ratio (95% CI)
		Territory	North	0.91 (0.89, 0.93)	Observed-to-expected ratio (95% CI)
		Territory	Northeast	0.90 (0.89, 0.91)	Observed-to-expected ratio (95% CI)
		Territory	Southeast	0.90 (0.90, 0.91)	Observed-to-expected ratio (95% CI)
		Territory	South	0.92 (0.91, 0.93)	Observed-to-expected ratio (95% CI)
		Territory	Midwest	0.89 (0.87, 0.91)	Observed-to-expected ratio (95% CI)
The US midlife mortality crisis continues: excess cause-specific mortality during 2020	Country, USA	Sex	Males	266	Excess deaths
		Sex	Females	−2429	Excess deaths
		Sex and age	M < 15	−24	Excess deaths
		Sex and age	M 15–24	9	Excess deaths
		Sex and age	M 25–44	41	Excess deaths
		Sex and age	M 45–64	672	Excess deaths
		Sex and age	M 65–74	73	Excess deaths
		Sex and age	M ≥ 75	−506	Excess deaths
		Sex and age	F < 15	−17	Excess deaths
		Sex and age	F 15–24	−5	Excess deaths
		Sex and age	F 25–44	−330	Excess deaths
		Sex and age	F 45–64	−821	Excess deaths
		Sex and age	F 65–74	675	Excess deaths
		Sex and age	F ≥ 75	−1931	Excess deaths
Child mortality in England during the first year of the COVID-19 pandemic	Country, UK	Aggregated		1.02 (0.86, 1.21)	Observed-to-expected ratio
Excess natural-cause deaths in California by cause and setting: March 2020 through February 2021	State, USA	Care setting	In hospital	−3,652 (−4,331, −2,968)	Excess deaths (95% CI)
		Care setting	Out of hospital	5,536 (5,090, 5,981)	Excess deaths (95% CI)

Considering deaths for diabetes (Table 4), Décarie and Michaud (2021) reported an increase in excess deaths in British Columbia (29.1), and a decrease in Quebec (−48.1), while Wu et al. (2021a, b) found more homogenous patterns of increased mortality for diabetes in several healthcare settings in England and Wales.

**Table 4:** Excess deaths from diabetes

Study title	Territory	Level of aggregation	Specific aggregation	Expected vs observed	Unit of measure
Excess mortality by specific causes of deaths in the city of São Paulo, Brazil, during the COVID-19 pandemic	City, San-Paolo, Brazil	Gender	Males	1.1 (0.52, 1.85)	Standardized mortality ratio (95% CI)
		Gender	Females	1.1 (0.47, 2.19)	Standardized mortality ratio (95% CI)
Counting the Counting the dead: COVID-19 and mortality in Quebec and British Columbia during the first wave	Territories, Canada	Territory	British Columbia	29.1	Excess deaths
		Territory	Quebec	−48.1	Excess deaths
Excess mortality in Wuhan city and other parts of China during the three months of the COVID-19 outbreak: findings from nationwide mortality registries	Territories, China	City	Wuhan	1.83 (1.08, 4.37)	Rate ratio (95% CI)
		Territory	Hubei without Wuhan	0.99 (0.72, 1.49)	Rate ratio (95% CI)
		Territory	China without Hubei	0.94 (0.84, 1.07)	Rate ratio (95% CI)
Temporal dynamic in the impact of COVID- 19 outbreak on cause-specific mortality in Guangzhou, China	City, China	Aggregated		10.2 (−3.7, 22.0)	% variation (95% CI)
		Gender	Males	13.5 (−1.4, 25.6)	% variation (95% CI)
		Gender	Females	6.7 (−9.2, 19.0)	% variation (95% CI)
		Age group	< 25	120.4 (−31.6, 182.8)	% variation (95% CI)
		Age group	25–44	2.3 (−33.2, 26.6)	% variation (95% CI)
		Age group	45–64	4.5 (−13.1, 16.9)	% variation (95% CI)
		Age group	65–74	6.7 (−10.5, 19.4)	% variation (95% CI)
		Age group	75–84	4.3 (−12.0, 16.5)	% variation (95% CI)
		Age group	85+	37.6 (19.7, 52.0)	% variation (95% CI)
		Setting of care	Hospital	2.4 (−24.3, 21.6)	% variation (95% CI)
		Setting of care	Outside hospitals	−19.1 (−46.1, −2.0)	% variation (95% CI)
		Marital status	Unmarried	−21.9 (−51.2, −1.9)	% variation (95% CI)
		Marital status	Married	−9.6 (−36.2, 6.9)	% variation (95% CI)
		Marital status	Divorced	−66.9 (−119.3, −35.9)	% variation (95% CI)
		Marital status	Widowed	62.6 (21.9, 89.0)	% variation (95% CI)
		Occupation class	Gold-collar	−24.5 (−84.8, 10.3)	% variation (95% CI)
		Occupation class	White-collar	7.7 (−39.4, 36.4)	% variation (95% CI)
		Occupation class	Pink-collar	-38.7 (−73.9, −14.8)	% variation (95% CI)
		Occupation class	Blue-collar	−1.7 (−25.5, 16.4)	% variation (95% CI)
		Occupation class	Others	−16.6 (−43.5, 2.4)	% variation (95% CI)
Place and underlying cause of death during the COVID-19 pandemic: retrospective cohort study of 3.5 million deaths in England and Wales, 2014 to 2020	Countries, England and Wales	Aggregated		683 (+32%)	Excess deaths (% variation)
		Setting of care	Home	296 (+52%)	Excess deaths (% variation)
		Setting of care	Care home or hospice	308 (+49%)	Excess deaths (% variation)
		Setting of care	Hospital	57 (+6%)	Excess deaths (% variation)
Variation in cause-specific mortality rates in Italy during the first wave of the COVID-19 pandemic: a study based on nationwide data	Country, Italy	Aggregated		32.6%	% variation
		Gender	Males	35.8%	% variation
		Gender	Females	29.3%	% variation
Leading causes of excess mortality in Mexico during the COVID-19 pandemic 2020–2021: A death certificates study in a middle-income country	Country, Mexico	Aggregated		80,294 (71,066, 89,522)	Excess deaths (95% CI)
Excess mortality associated with the COVID-19 pandemic in Latvia: a population-level analysis of all-cause and noncommunicable disease deaths in 2020	Country, Latvia	Aggregated		113 (−78, 353)	Excess deaths (95% CI)
Impact of the COVID-19 pandemic on total and cause-specific mortality in Pavia, Northern Italy	Province, Italy	Sex	Males	1.03 (0.82 ,1.30)	Standardized Mortality Ratio (95%CI)
		Sex	Females	1.13 (0.92, 1.40)	Standardized Mortality Ratio (95%CI)
		Sex and age	M 50–64		
		Sex and age	M 65–79		
		Sex and age	M 80+		
		Sex and age	F 50–64		
		Sex and age	F 65–79		
		Sex and age	F 80+		
The US midlife mortality crisis continues: excess cause-specific mortality during 2020	Country, USA	Sex	Males	7292	Excess deaths
		Sex	Females	6433	Excess deaths
		Sex and age	M < 15		Excess deaths
		Sex and age	M 15–24		Excess deaths
		Sex and age	M 25–44	535	Excess deaths
		Sex and age	M 45–64	2183	Excess deaths
		Sex and age	M 65–74	1947	Excess deaths
		Sex and age	M ≥ 75	2626	Excess deaths
		Sex and age	F < 15		Excess deaths
		Sex and age	F 15–24		Excess deaths
		Sex and age	F 25–44	279	Excess deaths
		Sex and age	F 45–64	1149	Excess deaths
		Sex and age	F 65–74	1551	Excess deaths
		Sex and age	F ≥ 75	3455	Excess deaths
Excess natural-cause deaths in California by cause and setting: March 2020 through February 2021	State, USA	Care setting	In hospital	51 (−143, 247)	Excess deaths (95% CI)
		Care setting	Out of hospital	2,050 (1,661, 2,443)	Excess deaths (95% CI)

Complete data for other causes of deaths can be found in the supplemental material (Tables S2–S26).

### Quality assessment

According to the Newcastle–Ottawa Quality Assessment Scale (Wells et al. 2022) (Table S18), most of the included articles showed a fair quality (*n* = 15; 68%, score = 7), and seven (32%) of the included articles showed a good quality (score = 8). None of the included studies showed high risk of bias.

## Discussion

### Main finding of this study

Investigating the true impact of the pandemic on causes of death other than COVID-19 is crucial for public health decision-making and defining priorities. In this study we systematically reviewed the body of literature on the patterns of cause-specific mortality during the COVID-19 pandemic.

The majority of the included studies analyzed study periods within the year 2020, while three of them also investigated 2021. This does not allow us to speculate about the long-term effects of the pandemic, nor on the impact of the vaccination campaign among the included countries. Conceivably, the effects on certain diseases could be delayed. Globally, the World Health Organization reported that 49% of countries experienced disruptions to diabetes services, 31% for cardiovascular disease services and 42% for cancer services due to the COVID-19 crisis (WHO 2020).

Considering that the types of studies were relatively similar (i.e., mainly cohort studies on excess mortality) and above all that the data used originated from national or supranational mortality registries characterized by controls and sufficient data security, the studies’ quality was high (between fair and good). The only substantial difference in the quality of the studies is related to the use of standardized mortality and the choice of controls for the implementation of the statistical models. This highlights how, despite the heterogeneity of the data sources and of the coding of the causes of death, the level of scientific evidence is high.

It should be noted that we could neither perform meta-analyses nor direct comparisons between studies due to the heterogeneity of methodologies and approaches. Specifically, authors investigated different time periods, adopted different methodologies for estimating excess mortality, different coding, and classification of causes of death, and, most importantly, they did not always standardize their results and adopted different approaches in the stratification of the results. Furthermore, despite the large body of literature on all-cause mortality, we found a relatively small number of studies focusing on specific causes of death.

Most of the studies included in this review reported excess all-cause mortality in the respective study periods, apart from Li et al. (2021) Kontopantelis et al. (2021), Glei (2022) and Chen et al. (2022). The scientific evidence accumulated so far confirms what has been reported by most of the studies included in our review, namely that the SARS-CoV-2 pandemic caused a global excess of deaths much greater than what is indicated by reported deaths due to COVID-19 alone (Wang et al. 2022; Glei 2022). The registered reduction of mortality in certain geographical areas and in certain care settings can be mainly explained by the measures adopted to counter the spread of the virus (i.e., social distancing, non-pharmaceutical interventions, mobility reduction, etc.). Notably, a reduction in hospice-related mortality also appears to be a common trend in recent pandemics (Bone et al. 2020; Chen et al. 2006).

Overall, there is still an important knowledge gap on cause-specific mortality patterns during the COVID-19 pandemic. In fact, most of the included studies investigated the causes of deaths that determine the greatest burden of disease in high-income countries (e.g., cardiovascular diseases, cancers, and diabetes). For instance, in the six Brazilian capitals analyzed by Brant et al. (2020), the impact of the pandemic on cardiovascular deaths was noticeable, especially in regions where the health system collapsed, which corresponds to the most socioeconomically deprived. This was also confirmed by Jardim et al. (2022). Li et al. (2021) also found excesses in deaths from cardiovascular diseases in China, particularly during the first wave of COVID-19, with an increase in observed deaths immediately after the start of the pandemic. Despite non-homogeneous results, an important share of the scientific literature reports an increase in cardiovascular diseases mortality during the first phases of the pandemic. Conceivably, the impaired access to healthcare services contributed to the increased cardiovascular mortality (Pines et al. 2021; De Luca et al. 2022; Perotti et al. 2022; Jardim et al. 2022; Glei 2022). Moreover, many cardiovascular diseases are time-dependent. An overwhelmed and fully committed health care system in the fight against respiratory disease caused by the SARS-CoV-2 pandemic partially neglected time-dependent conditions (Fox et al. 2022; Velek et al. 2022).

Although mortality patterns for cardiovascular diseases were very heterogeneous, for chronic diseases — such as oncological diseases — this aspect was even more marked. All the articles included in the review reported different results and addressed different aspects. For instance, opposite results were reported by Kontopantelis et al. (2021) on the one hand, who reported excess deaths for cancer during the first 30 weeks of the pandemic in England and Wales, and by Grande et al. (2022) on the other hand, who instead reported a fair percentage reduction in cancer mortality both overall and by gender during the first wave of the pandemic in Italy. This discrepancy can be explained by the different settings considered and by the different analytical approaches used in the articles. Nonetheless, the impact of the pandemic on the burden of cancer is still unknown and will probably be fully understood only in the next decades (Malagón et al. 2022; Englum et al. 2022). Moreover, the suspension of screening programs in many areas, combined with the reduction of surgical interventions and non-urgent visits, will affect cancer mortality, and increase its burden on societies, in a way which is still difficult to quantify.

When investigating mortality from road accidents, we discovered more homogeneous evidence. Most of the included studies (Liu et al. 2021; Li et al. 2021; Grande et al. 2022; Odd et al. 2022; Perotti et al. 2022) report a significant reduction in the number of road-accident deaths. By confining people at home, interrupting work activities, and reducing road traffic, the frequency of travel- and work-related conditions dropped. This is especially true for the first pandemic wave, when stricter confinement measures were adopted almost worldwide.

### What this study adds

Overall, the impact of the pandemic on non-COVID-19 cause-specific deaths has been very heterogeneous, and the analyses conducted so far are not exhaustive. Moreover, the current body of literature is limited in terms of number of studies and geographical location coverage. Specifically, the current evidence is limited to high-income and upper-middle-income countries only. This finding is a direct consequence of the lack of robust data management infrastructures and information systems and lack of reliable and up-to-date data in low-income countries (Lloyd-Sherlock et al. 2021). This lack of data provided by these countries is alarming because those countries are the ones with fewer resources to face the consequences of the pandemic (Bong et al. 2020), having also low supplies of SARS-CoV-2 vaccines (Mathieu et al. 2021). It is of utmost importance to invest in the improvement of infrastructures and information networks in low-income countries, to ensure updated and reliable data that would allow the rapid development of knowledge on which policymakers could base their decisions.

### Limitations of the study

This study faced some limitations that must be acknowledged. We were not able to compare the results of the studies, due to the differences in terms of study periods, adopted classification codes, stratification, and lack of standardization. The limited number of studies and the lack of representation of low- and middle-income countries did not make it possible to generalize the results. Moreover, the reduced number of studies that reported statistical significance and/or confidence intervals has further limited our ability to analyze the data. Another limitation is represented by misreporting and misclassification (Sanmarchi et al. 2021), especially for COVID-19 deaths in the early phases of the pandemic. This may have caused, especially in the most affected countries, an altered attribution of causes of death. Also, in this study we considered several subsequent pandemic waves with different variants of the SARS-CoV-2 lineage. Therefore, the impact of the virus on mortality could have been different, mainly due to differences in terms of intrinsic viral characteristics (i.e., pathogenicity and contagiousness). This may have at least partially influenced our findings. However, what we aimed to verify with this systematic review was not only the excess deaths that may have been caused directly by the virus and its pathogenicity, but also the indirect effects caused by the disruption of health services caused by the virus. Finally, when assessing the impact of the COVID-19 pandemic to develop and inform future public health strategies, it is important to analyze other consequences other than excess mortality only. COVID-19 can have long-term effects (Mathieu et al. 2021; Lopez-Leon et al. 2021) that can affect the quality of life of people who have contracted the disease. The emergency period affected people’s mental health (Adams-Prassl et al. 2022; Xie et al. 2022), and these effects will be quantifiable in the future, particularly for the younger generations. Future studies are needed to address these shortcomings.

## Conclusions

In this study, we reviewed the available literature to estimate the impact of the COVID-19 pandemic on different causes of death, and to provide a quantitative and qualitative analysis of the phenomenon. We found a high degree of heterogeneity of results and methodologies, which did not allow us to identify unique patterns of cause-specific mortality.

The SARS-CoV-2 pandemic wreaked havoc on society, economy, education, and health. At present, the extent of these effects is not completely known, and it will take time to fully understand them. We believe that it is of fundamental importance to produce novel and up-to-date evidence-based on robust and shared methods. Specifically, given the heterogeneity of the applied methodologies and the cause-specific mortality patterns detected, it would be appropriate to report standardized results (at least by age and sex) for specific macro-categories of death causes.

Reliable scientific evidence is needed by policymakers to make the best decisions in an unprecedented and extremely uncertain historical period, and this is true for the income level of all countries. We advocate for the urgent need to find an international consensus to define uniform conceptual and methodological approaches to establish the true burden of the COVID-19 pandemic on non-COVID-19 mortality.

## Supplementary information

ESM 1 (DOCX 186 kb)

## Data Availability

Data extracted from the included articles is available in the supplemental.
