# Supplementary material for: The impact of the SARS-CoV-2 pandemic on cause-specific mortality patterns: a systematic literature review
Source: Z Gesundh Wiss. 2022 Sep 26:1–19. Online ahead of print. doi: 10.1007/s10389-022-01755-7 (PMC9510758; doi:10.1007/s10389-022-01755-7)
Supplement: Supplementary file 1 — (DOCX 186 kb) [file 10389_2022_1755_MOESM1_ESM.docx]

**Supplemental**

**Table S1. Summary of the included studies**

| **Citation** | **Publication**  **Year** | **Territory** | **Income** | **Study**  **Period**  **start** | **Study**  **Period**  **end** | **Exected deaths methodology** | **Level of aggregation** | **Specific aggregation** | **All-cause excess mortality** | **Unit of measure** |
| --- | --- | --- | --- | --- | --- | --- | --- | --- | --- | --- |
| Brant LCC, Nascimento BR, Teixeira RA, et al. **Excess of cardiovascular deaths during the COVID-19 pandemic in Brazilian capital cities**. Heart. 2020;106(24):1898-1905. doi:10.1136/heartjnl-2020-317663 | 2020 | Country, Brazil | Upper-middle | 17/03/20 | 22/05/20 | Poisson regression models | Aggregated |  |  | % variation (95%CI) |
|  |  |  |  |  |  |  | City | São Paulo | 31.1(28.5;33.6) | % variation (95%CI) |
|  |  |  |  |  |  |  | City | Rio de Janeiro | 38.7(35.6;41.8) | % variation (95%CI) |
|  |  |  |  |  |  |  | City | Fortaleza | 87.7(80.8;94.8) | % variation (95%CI) |
|  |  |  |  |  |  |  | City | Recife | 71.7(64.3;79.3) | % variation (95%CI) |
|  |  |  |  |  |  |  | City | Belém | 126(114.6;138) | % variation (95%CI) |
|  |  |  |  |  |  |  | City | Manaus | 132.2(121.2;143.7) | % variation (95%CI) |
|  |  |  |  |  |  |  |  |  |  |  |
| Fernandes GA, Junior APN, Azevedo E Silva G, et al. **Excess mortality by specific causes of deaths in the city of São Paulo, Brazil, during the COVID-19 pandemic**. PLoS One. 2021;16(6):e0252238. Published 2021 Jun 7. doi:10.1371/journal.pone.0252238 | 2021 | City, Brazil | Upper-middle | 01/01/20 | 30/06/20 | Expected from 2019 | Aggregated |  |  | Standardized Mortality Ratio (95%CI) |
|  |  |  |  |  |  |  | Gender | Males | 1.3 1.17–1.42 | Standardized Mortality Ratio (95%CI) |
|  |  |  |  |  |  |  | Gender and age | M 0-9 | 0.8 0.46–1.31 | Standardized Mortality Ratio (95%CI) |
|  |  |  |  |  |  |  | Gender and age | M 10-29 | 1.3 0.69–2.19 | Standardized Mortality Ratio (95%CI) |
|  |  |  |  |  |  |  | Gender and age | M 30 - 44 | 1.4 0.92–2.11 | Standardized Mortality Ratio (95%CI) |
|  |  |  |  |  |  |  | Gender and age | M 45-59 | 1.3 1.04–1.66 | Standardized Mortality Ratio (95%CI) |
|  |  |  |  |  |  |  | Gender and age | M 65-79 | 1.3 1.16–1.52 | Standardized Mortality Ratio (95%CI) |
|  |  |  |  |  |  |  | Gender and age | M 80+ | 1.3 1.02–1.54 | Standardized Mortality Ratio (95%CI) |
|  |  |  |  |  |  |  | Gender | Females | 1.2 1.06–1.36 | Standardized Mortality Ratio (95%CI) |
|  |  |  |  |  |  |  | Gender and age | F 0-9 | 0.9 0.48–1.46 | Standardized Mortality Ratio (95%CI) |
|  |  |  |  |  |  |  | Gender and age | F 10-29 | 1.1 0.52–2.45 | Standardized Mortality Ratio (95%CI) |
|  |  |  |  |  |  |  | Gender and age | F 30 - 44 | 1.3 0.71–2.15 | Standardized Mortality Ratio (95%CI) |
|  |  |  |  |  |  |  | Gender and age | F 45-59 | 1.2 0.88–1.71 | Standardized Mortality Ratio (95%CI) |
|  |  |  |  |  |  |  | Gender and age | F 65-79 | 0 1.3 1.05–1.52 | Standardized Mortality Ratio (95%CI) |
|  |  |  |  |  |  |  | Gender and age | F 80+ | 1.2 0.89–1.45 | Standardized Mortality Ratio (95%CI) |
|  |  |  |  |  |  |  |  |  |  |  |
| Décarie Y, Michaud PC. **Counting the Dead: COVID-19 and Mortality in Quebec and British Columbia During the First Wave** [published online ahead of print, 2021 Sep 17]. Can Stud Popul. 2021;1-26. doi:10.1007/s42650-021-00053-z | 2021 | Territories, Canada | High | 01/03/20 | 30/06/2020 | Poisson regression models | Aggregated |  |  |  |
|  |  |  |  |  |  |  | Territory | British Columbia | 3921.6 (+13.4%) | excess deaths (% variation) |
|  |  |  |  |  |  |  | Territory | Quebec | 409.47 (+2.6%) | excess deaths (% variation) |
|  |  |  |  |  |  |  |  |  |  |  |
| Liu J, Zhang L, Yan Y, et al. **Excess mortality in Wuhan city and other parts of China during the three months of the covid-19 outbreak: findings from nationwide mortality registries**. BMJ. 2021;372:n415. Published 2021 Feb 24. doi:10.1136/bmj.n415 | 2021 | Territories, China | High | 01/01/2020 | 31/03/2020 | Farrington surveillance algorithm | Territory | Wuhan | 1.56 (1.33 to 1.87) | rate ratio (95%CI) |
|  |  |  |  |  |  |  | Territory | Hubei without Wuhan | 1.00 (0.86 to 1.16) | rate ratio (95%CI) |
|  |  |  |  |  |  |  | Territory | China without Hubei | 0.94 (0.86 to 1.04) | rate ratio (95%CI) |
|  |  |  |  |  |  |  |  |  |  |  |
| Li L, Hang D, Dong H, et al. **Temporal dynamic in the impact of COVID- 19 outbreak on cause-specific mortality in Guangzhou, China**. BMC Public Health. 2021;21(1):883. Published 2021 May 8. doi:10.1186/s12889-021-10771-3 | 2021 | City, China | High | 21/01/20 | 30/06/20 | Poisson regression models | Aggregated |  | − 4.1 (− 6.1, − 2.3) | % variation (95%CI) |
|  |  |  |  |  |  |  | Gender | Males | -5.2 (-7.3, -3.4) - | % variation (95%CI) |
|  |  |  |  |  |  |  | Gender | Female | -2.7 (-4.8, -0.7) | % variation (95%CI) |
|  |  |  |  |  |  |  | Age | <25 | -35.5 (-41.0, -30.4) | % variation (95%CI) |
|  |  |  |  |  |  |  | Age | 25-44 | -6.8 (-10.4, -3.4) | % variation (95%CI) |
|  |  |  |  |  |  |  | Age | 45-64 | -9.0 (-11.4, -6.8) | % variation (95%CI) |
|  |  |  |  |  |  |  | Age | 65-74 | 1.1 (-1.3, 3.5) | % variation (95%CI) |
|  |  |  |  |  |  |  | Age | 75-84 | -8.8 (-11.0, -6.6) | % variation (95%CI) |
|  |  |  |  |  |  |  | Age | 85+ | 4.0 (1.8, 6.2) | % variation (95%CI) |
|  |  |  |  |  |  |  | Place of death | In hospitals | -10.2 (-12.3, -8.0) | % variation (95%CI) |
|  |  |  |  |  |  |  | Place of death | Outside hospitals | -0.8 (-2.8, 1.3) | % variation (95%CI) |
|  |  |  |  |  |  |  | Marital status | Unmarried | -13.4 (-16.8, -10.1) | % variation (95%CI) |
|  |  |  |  |  |  |  | Marital status | Married | -6.0 (-8.1, -4.1) | % variation (95%CI) |
|  |  |  |  |  |  |  | Marital status | Divorced | -3.3 (-7.5, 0.8) | % variation (95%CI) |
|  |  |  |  |  |  |  | Marital status | Widowed | -1.3 (-3.5, 0.9) | % variation (95%CI) |
|  |  |  |  |  |  |  | Occupation class | Gold-collar | -18.7 (-23.9, -14.0) | % variation (95%CI) |
|  |  |  |  |  |  |  | Occupation class | White-collar | -15.0 (-18.9, -11.1) | % variation (95%CI) |
|  |  |  |  |  |  |  | Occupation class | Pink-collar | -12.1 (-16.3, -7.8) | % variation (95%CI) |
|  |  |  |  |  |  |  | Occupation class | Blue-collar | -3.6 (-5.7, -1.4) | % variation (95%CI) |
|  |  |  |  |  |  |  | Occupation class | Others | -5.1 (-7.1, -3.2) | % variation (95%CI) |
|  |  |  |  |  |  |  |  |  |  |  |
| Shoaib A, Van Spall HGC, Wu J, et al. **Substantial decline in hospital admissions for heart failure accompanied by increased community mortality during COVID-19 pandemic**. Eur Heart J Qual Care Clin Outcomes. 2021;7(4):378-387. doi:10.1093/ehjqcco/qcab040 | 2021 | Country, England | High | 01/02/20 | 31/05/20 | Mean | Aggregated |  |  |  |
|  |  |  |  |  |  |  | Care setting | Hospital |  |  |
|  |  |  |  |  |  |  | Care setting | Home |  |  |
|  |  |  |  |  |  |  | Care setting | Care homes and hospices |  |  |
|  |  |  |  |  |  |  |  |  |  |  |
| Wu J, Mafham M, Mamas MA, et al. **Place and Underlying Cause of Death During the COVID-19 Pandemic: Retrospective Cohort Study of 3.5 Million Deaths in England and Wales, 2014 to 2020**. Mayo Clin Proc. 2021;96(4):952-963. doi:10.1016/j.mayocp.2021.02.007 | 2021 | Countries, England and Wales | High | 02/03/20 | 30/06/20 | Poisson regression models | Aggregated | Aggregato | 57,860 (+35%) | total deaths (% variation) |
|  |  |  |  |  |  |  | Care setting | Home | 16,190 (+39%) | total deaths (% variation) |
|  |  |  |  |  |  |  | Care setting | Care home or hospice | 25,611 (+55%) | total deaths (% variation) |
|  |  |  |  |  |  |  | Care setting | Hospital | 15,938 (+21%) | total deaths (% variation) |
|  |  |  |  |  |  |  | Gender | Males | 29,956 (+36%) | total deaths (% variation) |
|  |  |  |  |  |  |  | Gender | Females | 27,839 (+33%) | total deaths (% variation) |
|  |  |  |  |  |  |  | Age | 18-49 | 982 (+15%) | total deaths (% variation) |
|  |  |  |  |  |  |  | Age | 50-59 | 2383 (+24%) | total deaths (% variation) |
|  |  |  |  |  |  |  | Age | 60-69 | 5251 (+28%) | total deaths (% variation) |
|  |  |  |  |  |  |  | Age | 70-79 | 12,017 (+31%) | total deaths (% variation) |
|  |  |  |  |  |  |  | Age | 80+ | 37,244 (+40%) | total deaths (% variation) |
|  |  |  |  |  |  |  | Territory | North East | 2563 (+27%) | total deaths (% variation) |
|  |  |  |  |  |  |  | Territory | North West | 7463 (+31%) | total deaths (% variation) |
|  |  |  |  |  |  |  | Territory | Yorkshire and The Humber | 4610 (+27%) | total deaths (% variation) |
|  |  |  |  |  |  |  | Territory | East Midlands | 3278 (+23%) | total deaths (% variation) |
|  |  |  |  |  |  |  | Territory | West Midlands | 6172 (+33%) | total deaths (% variation) |
|  |  |  |  |  |  |  | Territory | East of England | 4972 (+26%) | total deaths (% variation) |
|  |  |  |  |  |  |  | Territory | London | 9001 (+55%) | total deaths (% variation) |
|  |  |  |  |  |  |  | Territory | South East | 7026 (+26%) | total deaths (% variation) |
|  |  |  |  |  |  |  | Territory | South West | 2649 (+14%) | total deaths (% variation) |
|  |  |  |  |  |  |  | Territory | Wales | 1716 (+16%) | total deaths (% variation) |
|  |  |  |  |  |  |  |  |  |  |  |
| Al Wahaibi A, Al-Maani A, Alyaquobi F, et al. **Effects of COVID-19 on mortality: A 5-year population-based study in Oman**. Int J Infect Dis. 2021;104:102-107. doi:10.1016/j.ijid.2020.12.054 | 2021 | Country, Oman | High | 16/03/20 | 16/08/20 | Farrington surveillance algorithm | Aggregated |  | 15 (14–17) | Proportion of excess deaths (95% CI) |
|  |  |  |  |  |  |  | Care setting | Home | 7 (5–8) | Proportion of excess deaths (95% CI) |
|  |  |  |  |  |  |  | Care setting | Hospital | 12 (11–14) | Proportion of excess deaths (95% CI) |
|  |  |  |  |  |  |  |  |  |  |  |
| Wang J, Zhu J, Yang H, et al. **Cardiovascular-related deaths at the beginning of the COVID-19 outbreak: a prospective analysis based on the UK Biobank**. BMJ Open. 2021;11(6):e046931. Published 2021 Jun 4. doi:10.1136/bmjopen-2020-046931 | 2021 | Country, United Kingdom | High | 01/01/20 | 30/06/20 | Mean | Aggregated |  |  | Standardized Mortality Ratio [95%CI] |
|  |  |  |  |  |  |  | Time period | January | 0.94 [0.84 to 1.04] | Standardized Mortality Ratio [95%CI] |
|  |  |  |  |  |  |  | Time period | February | 1.05 [0.93 to 1.17] | Standardized Mortality Ratio [95%CI] |
|  |  |  |  |  |  |  | Time period | March | 1.30 [1.17 to 1.43] | Standardized Mortality Ratio [95%CI] |
|  |  |  |  |  |  |  | Time period | April | 1.51 [1.39 to 1.64] | Standardized Mortality Ratio [95%CI] |
|  |  |  |  |  |  |  | Time period | May | 0.84 [0.75 to 0.94] | Standardized Mortality Ratio [95%CI] |
|  |  |  |  |  |  |  | Time period | june | 1.03 [0.92 to 1.16] | Standardized Mortality Ratio [95%CI] |
|  |  |  |  |  |  |  |  |  |  |  |
| Wu J, Mamas MA, Mohamed MO, et al. **Place and causes of acute cardiovascular mortality during the COVID-19 pandemic**. Heart. 2021;107(2):113-119. doi:10.1136/heartjnl-2020-317912 | 2021 | Countries, England and Wales | High | 02/03/20 | 30/06/20 | Farrington surveillance algorithm | Aggregated |  |  |  |
|  |  |  |  |  |  |  | Gender | Males |  |  |
|  |  |  |  |  |  |  | Gender | Females |  |  |
|  |  |  |  |  |  |  | Age | 18–49 |  |  |
|  |  |  |  |  |  |  | Age | 50–59 |  |  |
|  |  |  |  |  |  |  | Age | 60-69 |  |  |
|  |  |  |  |  |  |  | Age | 70-79 |  |  |
|  |  |  |  |  |  |  | Age | 80+ |  |  |
|  |  |  |  |  |  |  | Care setting | Home |  |  |
|  |  |  |  |  |  |  | Care setting | Care home and hospice |  |  |
|  |  |  |  |  |  |  | Care setting | Hospital |  |  |
|  |  |  |  |  |  |  |  |  |  |  |
| Kontopantelis E, Mamas MA, Webb RT, et al. **Excess deaths from COVID-19 and other causes by region, neighbourhood deprivation level and place of death during the first 30 weeks of the pandemic in England and Wales: A retrospective registry study**. Lancet Reg Health Eur. 2021;7:100144. Published 2021 Jun 8. doi:10.1016/j.lanepe.2021.100144 | 2021 | Countries, England and Wales | High | 07/03/20 | 02/10/20 | Negative binomial regression models | Aggregated |  | 62,321 (58,849,65,793) | excess deaths (95%CI) |
|  |  |  |  |  |  |  | Gender | Males | 32,825 (31,529,34,121) | excess deaths (95%CI) |
|  |  |  |  |  |  |  | Gender | Females | 29,437 (28,150,30,724) | excess deaths (95%CI) |
|  |  |  |  |  |  |  | Age | 014 | -57 (-75,-39) | excess deaths (95%CI) |
|  |  |  |  |  |  |  | Age | 1544 | 534 (464,605) | excess deaths (95%CI) |
|  |  |  |  |  |  |  | Age | 4565 | 6925 (6686,7165) | excess deaths (95%CI) |
|  |  |  |  |  |  |  | Age | 6574 | 8596 (8270,8921) | excess deaths (95%CI) |
|  |  |  |  |  |  |  | Age | 7584 | 17,789 (17,214,18,364) | excess deaths (95%CI) |
|  |  |  |  |  |  |  | Age | 85+ | 22,146 (21,264,23,028) | excess deaths (95%CI) |
|  |  |  |  |  |  |  | Territory | North East | 3303 (3220,3386) | excess deaths (95%CI) |
|  |  |  |  |  |  |  | Territory | North West | 8782 (8579,8985) | excess deaths (95%CI) |
|  |  |  |  |  |  |  | Territory | Yorkshire & Humber | 5295 (5145,5446) | excess deaths (95%CI) |
|  |  |  |  |  |  |  | Territory | East Midlands | 4750 (4621,4879) | excess deaths (95%CI) |
|  |  |  |  |  |  |  | Territory | West Midlands | 7730 (7564,7896) | excess deaths (95%CI) |
|  |  |  |  |  |  |  | Territory | East of England | 5995 (5821,6169) | excess deaths (95%CI) |
|  |  |  |  |  |  |  | Territory | London | 10,311 (10,166,10,456) | excess deaths (95%CI) |
|  |  |  |  |  |  |  | Territory | South East Coast | 5532 (5395,5669) | excess deaths (95%CI) |
|  |  |  |  |  |  |  | Territory | South Central | 4043 (3938,4147) | excess deaths (95%CI) |
|  |  |  |  |  |  |  | Territory | South West | 4410 (4243,4577) | excess deaths (95%CI) |
|  |  |  |  |  |  |  | Territory | Wales | 2546 (2448,2644) | excess deaths (95%CI) |
|  |  |  |  |  |  |  | Deprivation quintiles | 1 (least deprived) | 10,770 (10,427,11,113) | excess deaths (95%CI) |
|  |  |  |  |  |  |  | Deprivation quintiles | 2 | 11,414 (11,046,11,782) | excess deaths (95%CI) |
|  |  |  |  |  |  |  | Deprivation quintiles | 3 | 12,649 (12,279,13,018) | excess deaths (95%CI) |
|  |  |  |  |  |  |  | Deprivation quintiles | 4 | 13,053 (12,698,13,408) | excess deaths (95%CI) |
|  |  |  |  |  |  |  | Deprivation quintiles | 5 (most deprived) | 14,789 (14,424,15,154) | excess deaths (95%CI) |
|  |  |  |  |  |  |  | Place of death | Care home | 23,280 (22,778,23,781) | excess deaths (95%CI) |
|  |  |  |  |  |  |  | Place of death | Home | 26,141 (25,633,26,650) | excess deaths (95%CI) |
|  |  |  |  |  |  |  | Place of death | Hospice | -1603 (-1734,-1473) | excess deaths (95%CI) |
|  |  |  |  |  |  |  | Place of death | Hospital | 10,034 (9060,11,008) | excess deaths (95%CI) |
|  |  |  |  |  |  |  | Place of death | Other/ Unknown | 1565 (1499,1632) | excess deaths (95%CI) |
|  |  |  |  |  |  |  |  |  |  |  |
| Sharma R, Kuohn LR, Weinberger DM, et al. **Excess Cerebrovascular Mortality in the United States During the COVID-19 Pandemic**. Stroke. 2021;52(2):563-572. doi:10.1161/STROKEAHA.120.031975 | 2021 | States, USA | High | 01/01/20 | 16/05/20 | Poisson regression models | Aggregated |  |  |  |
|  |  |  |  |  |  |  |  |  |  |  |
| Faust JS, Du C, Mayes KD, et al. **Mortality From Drug Overdoses, Homicides, Unintentional Injuries, Motor Vehicle Crashes, and Suicides During the Pandemic**, March-August 2020. JAMA. 2021;326(1):84-86. doi:10.1001/jama.2021.8012 | 2021 | Country, USA | High | 01/01/20 | 31/08/20 | seasonal autore-gressive integrated moving average (sARIMA) model | Aggregated |  | 1.18 (1.11-1.27) | observed-to-expected ratio (95%CI) |
|  |  |  |  |  |  |  | Time period | March |  | observed-to-expected ratio (95%CI) |
|  |  |  |  |  |  |  | Time period | April |  | observed-to-expected ratio (95%CI) |
|  |  |  |  |  |  |  | Time period | May |  | observed-to-expected ratio (95%CI) |
|  |  |  |  |  |  |  | Time period | June |  | observed-to-expected ratio (95%CI) |
|  |  |  |  |  |  |  | Time period | July |  | observed-to-expected ratio (95%CI) |
|  |  |  |  |  |  |  | Time period | August |  | observed-to-expected ratio (95%CI) |
|  |  |  |  |  |  |  |  |  |  |  |
| Grande E, Fedeli U, Pappagallo M, et al. **Variation in Cause-Specific Mortality Rates in Italy during the First Wave of the COVID-19 Pandemic: A Study Based on Nationwide Data**. Int J Environ Res Public Health. 2022;19(2):805. Published 2022 Jan 12. doi:10.3390/ijerph19020805 | 2022 | Country, Italy | High | 01/03/20 | 30/04/20 | mean | Aggregated |  | 36.3% | % variation |
|  |  |  |  |  |  |  | Gender | Males | 39.0% | % variation |
|  |  |  |  |  |  |  | Gender | Females | 31.9% | % variation |
| Palacio-Mejía LS, Hernández-Ávila JE, Hernández-Ávila M, et al. **Leading causes of excess mortality in Mexico during the COVID-19 pandemic 2020-2021: A death certificates study in a middle-income country.** Lancet Reg Health Am. 2022;13:100303. doi:10.1016/j.lana.2022.100303 | 2022 | Country, Mexico | Upper-middle | 01/01/20 | 31/12/21 | Poisson regression models | Aggregated |  | 600,592 (575,125 to 626,056) | excess deaths (95%CI) |
| Gobiņa I, Avotiņš A, Kojalo U, et al. **Excess mortality associated with the COVID-19 pandemic in Latvia: a population-level analysis of all-cause and noncommunicable disease deaths in 2020**. BMC Public Health. 2022;22(1):1109. Published 2022 Jun 3. doi:10.1186/s12889-022-13491-4 | 2022 | Country, Latvia | High | 01/04/20 | 29/12/20 | Generalised additive modelling (GAM) | Aggregated |  | 3111 (1339 – 4832) | excess deaths (95%CI) |
| Perotti P, Bertuccio P, Cacitti S, et al**. Impact of the COVID-19 Pandemic on Total and Cause-Specific Mortality in Pavia, Northern Italy.** Int J Environ Res Public Health. 2022;19(11):6498. Published 2022 May 26. doi:10.3390/ijerph19116498 | 2022 | Province, Italy | High | 1/1/20 | 31/12/20 | Mean | Sex | Males | 1.24 (1.20-1.28) | Standardized Mortality Ratio (95%CI) |
|  |  |  |  |  |  |  | Sex | Females | 1.25 (1.21-1.30) | Standardized Mortality Ratio (95%CI) |
|  |  |  |  |  |  |  | Sex and Age | M 50-64 |  |  |
|  |  |  |  |  |  |  | Sex and Age | M 65-79 |  |  |
|  |  |  |  |  |  |  | Sex and Age | M 80+ |  |  |
|  |  |  |  |  |  |  | Sex and Age | F 50-64 |  |  |
|  |  |  |  |  |  |  | Sex and Age | F 65-79 |  |  |
|  |  |  |  |  |  |  | Sex and Age | F 80+ |  |  |
| Jardim BC, Migowski A, Corrêa FM, Silva GAE. **Covid-19 in Brazil in 2020: impact on deaths from cancer and cardiovascular diseases.** Rev Saude Publica. 2022;56:22. Published 2022 Apr 22. doi:10.11606/s1518-8787.2022056004040 | 2022 | Country, Brasil | Upper-middle | 1/3/20 | 31/12/20 | Poisson regression models | Aggregated |  |  |  |
|  |  |  |  |  |  |  | Territory | North |  |  |
|  |  |  |  |  |  |  | Territory | Northeast |  |  |
|  |  |  |  |  |  |  | Territory | Southeast |  |  |
|  |  |  |  |  |  |  | Territory | South |  |  |
|  |  |  |  |  |  |  | Territory | Midwest |  |  |
|  | 2022 | Country, Brasil | Upper-middle | 1/3/20 | 31/12/20 | Poisson regression models | Aggregated |  |  |  |
| Glei DA. **The US Midlife Mortality Crisis Continues: Excess Cause-Specific Mortality During 2020** [published online ahead of print, 2022 Mar 24]. Am J Epidemiol. 2022;kwac055. doi:10.1093/aje/kwac055 | 2022 | Country, USA | High | 1/3/20 | 31/12/20 | Negative binomial regression models | Sex | Males | 252,199 | excess deaths |
|  |  |  |  |  |  |  | Sex | Females | 196,481 | excess deaths |
|  |  |  |  |  |  |  | Sex and Age | M <15 | –446 | excess deaths |
|  |  |  |  |  |  |  | Sex and Age | M 15-24 | 4,495 | excess deaths |
|  |  |  |  |  |  |  | Sex and Age | M 25-44 | 21300 | excess deaths |
|  |  |  |  |  |  |  | Sex and Age | M 45-64 | 64,862 | excess deaths |
|  |  |  |  |  |  |  | Sex and Age | M 65 - 74 | 54,284 | excess deaths |
|  |  |  |  |  |  |  | Sex and Age | M >=75 | 107,703 | excess deaths |
|  |  |  |  |  |  |  | Sex and Age | F <15 | –798 | excess deaths |
|  |  |  |  |  |  |  | Sex and Age | F 15-24 | 1,113 | excess deaths |
|  |  |  |  |  |  |  | Sex and Age | F 25-44 | 8260 | excess deaths |
|  |  |  |  |  |  |  | Sex and Age | F 45-64 | 29,047 | excess deaths |
|  |  |  |  |  |  |  | Sex and Age | F 65 - 74 | 36,815 | excess deaths |
|  |  |  |  |  |  |  | Sex and Age | F >=75 | 122,045 | excess deaths |
| Orellana JDY, de Souza MLP. **Excess suicides in Brazil: Inequalities according to age groups and regions during the COVID-19 pandemic.** Int J Soc Psychiatry. 2022;68(5):997-1009. doi:10.1177/00207640221097826 | 2022 | Country, Brasil | Upper-middle | 01/08/2020 | 31/12/2020 | Generalised additive modelling (GAM) | Aggregated |  |  |  |
|  |  |  |  |  |  |  | Age | 10-29 |  |  |
|  |  |  |  |  |  |  | Age | 30-59 |  |  |
|  |  |  |  |  |  |  | Age | >=60 |  |  |
|  |  |  |  |  |  |  | Sex | Females |  |  |
|  |  |  |  |  |  |  | Sex and Age | 10-29 |  |  |
|  |  |  |  |  |  |  | Sex and Age | 30-59 |  |  |
|  |  |  |  |  |  |  | Sex and Age | >=60 |  |  |
|  |  |  |  |  |  |  | Sex | Males |  |  |
|  |  |  |  |  |  |  | Sex and Age | 10-29 |  |  |
|  |  |  |  |  |  |  | Sex and Age | 30-59 |  |  |
|  |  |  |  |  |  |  | Sex and Age | >=60 |  |  |
| Odd D, Stoianova S, Williams T, Fleming P, Luyt K. **Child mortality in England during the first year of the COVID-19 pandemic**. Arch Dis Child. 2022;107(3):e22. doi:10.1136/archdischild-2021-323370 | 2022 | Country, UK | High | 01/04/2020 | 31/03/2021 | Negative Binomial Regression model | Aggregated |  |  | observed-to-expected ratio (95%CI) |
| Chen YH, Stokes AC, Aschmann HE, et al**. Excess natural-cause deaths in California by cause and setting: March 2020 through February 2021.** PNAS Nexus. 2022;1(3):pgac079. Published 2022 Jun 8. doi:10.1093/pnasnexus/pgac079 | 2022 | State, USA | High | 01/03/2020 | 28/02/2021 | Autoregressive integrated moving average (ARIMA) | Care setting | In hospital | 36,407 (34,718 to 38,096) | excess deaths (95%CI) |
|  |  |  |  |  |  |  | Care setting | Out of hospital | 32,775 (29,509 to 36,082) | excess deaths (95%CI) |
|  |  |  |  |  |  |  |  |  |  |  |

**INSERISCI QUI LE ALTRE**

**Table S2. Cardiovascular diseases**

| **Study title** | **Territory** | **Level of aggregation** | **Specific aggregation** | **Expected vs observed** | **Unit of measure** |
| --- | --- | --- | --- | --- | --- |
| Excess of cardiovascular deaths during the COVID-19 pandemic in Brazilian capital cities | Country, Brazil | City | São Paulo | 10.1% (5.2, 15.3) | % variation (95%CI) |
|  |  | City | Rio de Janeiro | -7.1% (-11.9, -1.9) | % variation (95%CI) |
|  |  | City | Fortaleza | 12.6% (2.4, 23.8) | % variation (95%CI) |
|  |  | City | Recife | 6.6% (-4.4, 18.8) | % variation (95%CI) |
|  |  | City | Belém | 43.6% (27.3, 62) | % variation (95%CI) |
|  |  | City | Manaus | 46.1% (29.5, 64.9) | % variation (95%CI) |
| Excess mortality by specific causes of deaths in the city of São Paulo, Brazil, during the COVID-19 pandemic | City, San-Paolo, Brazil | Gender | Males | 0.9 (0.75, 1.13) | Standardized Mortality Ratio (95%CI) |
|  |  | Gender | Females | 0.9 (0.68, 1.15) | Standardized Mortality Ratio (95%CI) |
| Counting the Dead: COVID-19 and Mortality in Quebec and British Columbia During the First Wave | Territories, Canada | Territory | British Columbia | -67.1 | Excess deaths |
|  |  | Territory | Quebec | -279.0 | Excess deaths |
| Excess mortality in Wuhan city and other parts of China during the three months of the covid-19 outbreak: findings from nationwide mortality registries | Territories, China | City | Wuhan | 1.29 (1.05, 1.65) | Rate ratio (95%CI) |
|  |  | Territory | Hubei without Wuhan | 0.98 (0.83, 1.18) | Rate ratio (95%CI) |
|  |  | Territory | China without Hubei | 0.95 (0.86, 1.07) | Rate ratio (95%CI) |
| Temporal dynamic in the impact of COVID- 19 outbreak on cause-specific mortality in Guangzhou, China | City, China | Aggregated |  | 1.9 (-1.5, 5.0) | % variation (95%CI) |
|  |  | Gender | Males | 1.6 (-1.9, 4.6) | % variation (95%CI) |
|  |  | Gender | Females | 2.3 (-0.8, 5.4) | % variation (95%CI) |
|  |  | Age group | <25 | -24.4 (-46.5, -5.3) | % variation (95%CI) |
|  |  | Age group | 25-44 | 12.9 (4.6, 20.5) | % variation (95%CI) |
|  |  | Age group | 45-64 | -9.6 (-13.5, -6.0) | % variation (95%CI) |
|  |  | Age group | 65-74 | 5.3 (1.6, 9.0) | % variation (95%CI) |
|  |  | Age group | 75-84 | -3.5 (-7.0, -0.2) | % variation (95%CI) |
|  |  | Age group | 85+ | 11.1 (7.8, 14.2) | % variation (95%CI) |
|  |  | Setting of care | Hospital | -3.8 (-7.4, -0.5) | % variation (95%CI) |
|  |  | Setting of care | Outside hospitals | 3.6 (0.4, 6.9) | % variation (95%CI) |
|  |  | Marital status | Unmarried | 13.5 (7.1, 18.9) | % variation (95%CI) |
|  |  | Marital status | Married | -1.7 (-5.1, 1.5) | % variation (95%CI) |
|  |  | Marital status | Divorced | 4.2 (-3.2, 10.7) | % variation (95%CI) |
|  |  | Marital status | Widowed | 4.0 (0.3, 7.2) | % variation (95%CI) |
|  |  | Occupation class | Gold-collar | -15.5 (-25.9, -7.1) | % variation (95%CI) |
|  |  | Occupation class | White-collar | -10.3 (-18.5, -3.0) | % variation (95%CI) |
|  |  | Occupation class | Pink-collar | 3.8 (-4.5, 10.7) | % variation (95%CI) |
|  |  | Occupation class | Blue-collar | -3.1 (-6.5, -0.2) | % variation (95%CI) |
|  |  | Occupation class | Others | 3.8 (0.7, 6.8) | % variation (95%CI) |
| Substantial decline in hospital admissions for heart failure accompanied by increased community mortality during COVID-19 pandemic | Country, England | Setting of care | Hospital | 0.71 (-0.08, -1.23) | Incidence rate ratio (95%CI) |
|  |  | Setting of care | Home | 1.31 (1.24, 1.39) | Incidence rate ratio (95%CI) |
|  |  | Setting of care | Care homes and hospices | 1.28 (1.18, 1.40) | Incidence rate ratio (95%CI) |
| Place and Underlying Cause of Death During the COVID-19 Pandemic: Retrospective Cohort Study of 3.5 Million Deaths in England and Wales, 2014 to 2020 | Countries, England and Wales | Aggregated |  | 2225 (+9%) | Excess deaths (% variation) |
|  |  | Setting of care | Home | 2485 (+26%) | Excess deaths (% variation) |
|  |  | Setting of care | Care home or hospice | 1211 (+31%) | Excess deaths (% variation) |
|  |  | Setting of care | Hospital | -1398 (-13%) | Excess deaths (% variation) |
| Effects of COVID-19 on mortality: A 5-year population-based study in Oman | Country, Oman | Aggregated |  | 0 | Observed-to-expected ratio (95%CI) |
|  |  | Setting of care | Home | 0 | Observed-to-expected ratio (95%CI) |
|  |  | Setting of care | hospital | 0 | Observed-to-expected ratio (95%CI) |
| Cardiovascular-related deaths at the beginning of the COVID-19 outbreak: a prospective analysis based on the UK Biobank | Country, Oman | Time period | March | 1.19 (1.00, 1.40) | Standardized Mortality Ratio (95%CI) |
|  |  | Time period | April | 0.98 (0.81, 1.17) | Standardized Mortality Ratio (95%CI) |
|  |  | Time period | May | 0.67 (0.54, 0.83) | Standardized Mortality Ratio (95%CI) |
|  |  | Time period | June | 0.87 (0.70, 1.06) | Standardized Mortality Ratio (95%CI) |
| Place and causes of acute cardiovascular mortality during the COVID-19 pandemic | Countries, England and Wales | Aggregated |  | 2085 (+8%) | Excess deaths (% variation) |
|  |  | Gender | Males | 1182 (+8%) | Excess deaths (% variation) |
|  |  | Gender | Females | 948 (+7%) | Excess deaths (% variation) |
|  |  | Age | 18–49 | 176 (+17%) | Excess deaths (% variation) |
|  |  | Age | 50–59 | 248 (+14%) | Excess deaths (% variation) |
|  |  | Age | 60-69 | 468 (+15%) | Excess deaths (% variation) |
|  |  | Age | 70-79 | 688 (+11%) | Excess deaths (% variation) |
|  |  | Age | 80+ | 734 (+5%) | Excess deaths (% variation) |
|  |  | Setting of care | Home | 2279 (+35%) | Excess deaths (% variation) |
|  |  | Setting of care | Care home and hospice | 1095 (+32%) | Excess deaths (% variation) |
|  |  | Setting of care | Hospital | 50 (0%) | Excess deaths (% variation) |
| Excess Cerebrovascular Mortality in the United States During the COVID-19 Pandemic | States, USA | Aggregated |  | 6367 | Excess deaths |
| Excess mortality associated with the COVID-19 pandemic in Latvia: a population-level analysis of all-cause and noncommunicable disease deaths in 2020 | Country, Latvia | Aggregated |  | 1309 (88, 2476) | Excess deaths (% variation) |
| Impact of the COVID-19 Pandemic on Total and Cause-Specific Mortality in Pavia, Northern Italy | Province, Italy | Sex | Males | 0.89 (0.83, 0.96) | Observed-to-expected ratio (95%CI) |
|  |  | Sex | Females | 0.96 (0.90, 1.01) | Observed-to-expected ratio (95%CI) |
|  |  | Sex and Age | M 50-64 | 0.93 (0.72, 10.2) | Observed-to-expected ratio (95%CI) |
|  |  | Sex and Age | M 65-79 | 1.00 (0.84, 1.19) | Observed-to-expected ratio (95%CI) |
|  |  | Sex and Age | M 80+ | 0.83 (0.76, 0.91) | Observed-to-expected ratio (95%CI) |
|  |  | Sex and Age | F 50-64 | 1.06 (0.69, 1.63) | Observed-to-expected ratio (95%CI) |
|  |  | Sex and Age | F 65-79 | 1.01 (0.83, 1.22) | Observed-to-expected ratio (95%CI) |
|  |  | Sex and Age | F 80+ | 0.95 (0.89, 1.00) | Observed-to-expected ratio (95%CI) |
| Covid-19 in Brazil in 2020: impact on deaths from cancer and cardiovascular diseases | Country, Brasil | Aggregated |  | 0.90 (0.90, 0.91) | Observed-to-expected ratio (95%CI) |
|  |  | Territory | North | 0.91 (0.89, 0.93) | Observed-to-expected ratio (95%CI) |
|  |  | Territory | Northeast | 0.90 (0.89, 0.91) | Observed-to-expected ratio (95%CI) |
|  |  | Territory | Southeast | 0.90 (0.90, 0.91) | Observed-to-expected ratio (95%CI) |
|  |  | Territory | South | 0.92 (0.91, 0.93) | Observed-to-expected ratio (95%CI) |
|  |  | Territory | Midwest | 0.89 (0.87, 0.91) | Observed-to-expected ratio (95%CI) |
| The US Midlife Mortality Crisis Continues: Excess Cause-Specific Mortality During 2020 | Country, USA | Sex | Males | 15943 | Excess deaths |
|  |  | Sex | Females | 10566 | Excess deaths |
|  |  | Sex and Age | M <15 | - | Excess deaths |
|  |  | Sex and Age | M 15-24 | - | Excess deaths |
|  |  | Sex and Age | M 25-44 | 1589 | Excess deaths |
|  |  | Sex and Age | M 45-64 | 7122 | Excess deaths |
|  |  | Sex and Age | M 65 - 74 | 2534 | Excess deaths |
|  |  | Sex and Age | M >=75 | 4698 | Excess deaths |
|  |  | Sex and Age | F <15 | - | Excess deaths |
|  |  | Sex and Age | F 15-24 | 21 | Excess deaths |
|  |  | Sex and Age | F 25-44 | 607 | Excess deaths |
|  |  | Sex and Age | F 45-64 | 2110 | Excess deaths |
|  |  | Sex and Age | F 65 - 74 | 2294 | Excess deaths |
|  |  | Sex and Age | F >=75 | 5535 | Excess deaths |
| Excess natural-cause deaths in California by cause and setting: March 2020 through February 2021 | State, USA | Care setting | In hospital | −1,930 (−2,520, −1,336) | Excess deaths (95%CI) |
|  |  | Care setting | Out of hospital | 7,649 (5,762, 9,539) | Excess deaths (95%CI) |

**Table S3. Cancers**

| **Study title** | **Territory** | **Level of aggregation** | **Specific aggregation** | **Expected vs observed** | **Unit of measure** |
| --- | --- | --- | --- | --- | --- |
| Excess mortality by specific causes of deaths in the city of São Paulo, Brazil, during the COVID-19 pandemic | City, San-Paolo, Brazil | Gender | Males | 0.9 (0.66, 1.09) | Standardized Mortality Ratio (95%CI) |
|  |  | Gender | Females | 0.9 (0.67, 1.20) | Standardized Mortality Ratio (95%CI) |
| Counting the Dead: COVID-19 and Mortality in Quebec and British Columbia During the First Wave | Territories, Canada | Territory | British Columbia | -45.0 | Excess deaths |
|  |  | Territory | Quebec | -570.5 | Excess deaths |
| Excess mortality in Wuhan city and other parts of China during the three months of the covid-19 outbreak: findings from nationwide mortality registries | Territories, China | City | Wuhan | 1.02 (0.81, 1.33) | Rate ratio (95%CI) |
|  |  | Territory | Hubei without Wuhan | 1.08 (0.94, 1.25) | Rate ratio (95%CI) |
|  |  | Territory | China without Hubei | 0.99 (0.93, 1.06) | Rate ratio (95%CI) |
| Temporal dynamic in the impact of COVID- 19 outbreak on cause-specific mortality in Guangzhou, China | City, China | Aggregated |  | 1.3 (-2.8, 4.8) | % variation (95%CI) |
|  |  | Gender | Males | 1.4 (-2.4, 5.1) | % variation (95%CI) |
|  |  | Gender | Females | 1.0 (-3.0, 4.6) | % variation (95%CI) |
|  |  | Age group | <25 | -13.6 (-29.4, -0.4) | % variation (95%CI) |
|  |  | Age group | 25-44 | 1.7 (-4.5, 7.5) | % variation (95%CI) |
|  |  | Age group | 45-64 | 0.6 (-3.7, 4.6) | % variation (95%CI) |
|  |  | Age group | 65-74 | 8.6 (4.2, 12.7) | % variation (95%CI) |
|  |  | Age group | 75-84 | -4.7 (-9.1, -0.5) | % variation (95%CI) |
|  |  | Age group | 85+ | 0.2 (-4.9, 5.2) | % variation (95%CI) |
|  |  | Setting of care | Hospital | -5.4 (-9.5, -1.8) | % variation (95%CI) |
|  |  | Setting of care | Outside hospitals | 7.6 (3.6, 11.2) | % variation (95%CI) |
|  |  | Marital status | Unmarried | -1.3 (-8.9, 5.3) | % variation (95%CI) |
|  |  | Marital status | Married | -0.2 (-4.0, 3.4) | % variation (95%CI) |
|  |  | Marital status | Divorced | 10.8 (2.8, 17.7) | % variation (95%CI) |
|  |  | Marital status | Widowed | -0.1 (-4.7, 4.6) | % variation (95%CI) |
|  |  | Occupation class | Gold-collar | -19.0 (-27.8, -11.1) | % variation (95%CI) |
|  |  | Occupation class | White-collar | -8.1 (-14.9, -1.9) | % variation (95%CI) |
|  |  | Occupation class | Pink-collar | -12.2 (-19.2, -5.6) | % variation (95%CI) |
|  |  | Occupation class | Blue-collar | 3.8 (-0.8, 7.7) | % variation (95%CI) |
|  |  | Occupation class | Others | 0.3 (-3.8, 3.9) | % variation (95%CI) |
| Place and Underlying Cause of Death During the COVID-19 Pandemic: Retrospective Cohort Study of 3.5 Million Deaths in England and Wales, 2014 to 2020 | Countries, England and Wales | Aggregated |  | 687 (+1%) | Excess deaths (% variation) |
|  |  | Setting of care | Home | 5963 (+40%) | Excess deaths (% variation) |
|  |  | Setting of care | Care home or hospice | -1495 (-10%) | Excess deaths (% variation) |
|  |  | Setting of care | Hospital | -4088 (-24%) | Excess deaths (% variation) |
| Excess deaths from COVID-19 and other causes by region, neighbourhood deprivation level and place of death during the first 30 weeks of the pandemic in England and Wales: A retrospective registry study | Countries, England and Wales | Aggregated |  | 1668 (289, 3047) | Excess deaths (95%CI) |
|  |  | Gender | Males | 857 (531, 1183) | Excess deaths (95%CI) |
|  |  | Gender | Females | 812 (527, 1097) | Excess deaths (95%CI) |
|  |  | Age group | 0-14 | -5 (-9, -1) | Excess deaths (95%CI) |
|  |  | Age group | 15-44 | 20 (6, 34) | Excess deaths (95%CI) |
|  |  | Age group | 45-65 | 417 (329, 505) | Excess deaths (95%CI) |
|  |  | Age group | 65-74 | 486 (358, 615) | Excess deaths (95%CI) |
|  |  | Age group | 75-84 | 681 (517, 845) | Excess deaths (95%CI) |
|  |  | Age group | 85+ | -500 (-621, -378) | Excess deaths (95%CI) |
|  |  | Territory | North East | 67 (39, 94) | Excess deaths (95%CI) |
|  |  | Territory | North West | 168 (107, 228) | Excess deaths (95%CI) |
|  |  | Territory | Yorkshire & Humber | 128 (79, 176) | Excess deaths (95%CI) |
|  |  | Territory | East Midlands | 203 (160, 247) | Excess deaths (95%CI) |
|  |  | Territory | West Midlands | 293 (242, 344) | Excess deaths (95%CI) |
|  |  | Territory | East of England | -39 (-92, 14) | Excess deaths (95%CI) |
|  |  | Territory | London | 116 (71, 162) | Excess deaths (95%CI) |
|  |  | Territory | South East Coast | 359 (316, 402) | Excess deaths (95%CI) |
|  |  | Territory | South Central | 147 (110, 183) | Excess deaths (95%CI) |
|  |  | Territory | South West | 327 (274, 380) | Excess deaths (95%CI) |
|  |  | Territory | Wales | -30 (-62, 2) | Excess deaths (95%CI) |
|  |  | Deprivation quintiles | 1 (least deprived) | 453 (357, 549) | Excess deaths (95%CI) |
|  |  | Deprivation quintiles | 2 | -71 (31, 172) | Excess deaths (95%CI) |
|  |  | Deprivation quintiles | 3 | 228 (128, 328) | Excess deaths (95%CI) |
|  |  | Deprivation quintiles | 4 | 446 (353, 538) | Excess deaths (95%CI) |
|  |  | Deprivation quintiles | 5 (most deprived) | 542 (448, 635) | Excess deaths (95%CI) |
|  |  | Setting of care | Care home | -917 (-1000, -835) | Excess deaths (95%CI) |
|  |  | Setting of care | Home | 10665 (10498, 10833) | Excess deaths (95%CI) |
|  |  | Setting of care | Hospice | -2186 (-2278,-2094) | Excess deaths (95%CI) |
|  |  | Setting of care | Hospital | -6655 (-6854,-6456) | Excess deaths (95%CI) |
|  |  | Setting of care | Other/ Unknown | 749 (735, 762) | Excess deaths (95%CI) |
| Variation in Cause-Specific Mortality Rates in Italy during the First Wave of the COVID-19 Pandemic: A Study Based on Nationwide Data | Country, Italy | Aggregated |  | -4.3% | % variation |
|  |  | Gender | Males | -5.7% | % variation |
|  |  | Gender | Females | -2.9% | % variation |
| Leading causes of excess mortality in Mexico during the COVID-19 pandemic 2020-2021: A death certificates study in a middle-income country | Country, Mexico | Aggregated |  | -10,126 (-19,044, -1209) | Excess deaths (95%CI) |
| Excess mortality associated with the COVID-19 pandemic in Latvia: a population-level analysis of all-cause and noncommunicable disease deaths in 2020 | Country, Latvia | Aggregated |  | 208 (-656, 1025) | Excess deaths (95%CI) |
| Impact of the COVID-19 Pandemic on Total and Cause-Specific Mortality in Pavia, Northern Italy | Province, Italy | Sex | Males | 0.86 (0.80, 0.92) | Standardized Mortality Ratio (95%CI) |
|  |  | Sex | Females | 0.97 (0.90, 1.04) | Standardized Mortality Ratio (95%CI) |
|  |  | Sex and Age | M 50-64 | 0.77 (0.65, 0.92) | Standardized Mortality Ratio (95%CI) |
|  |  | Sex and Age | M 65-79 | 0.85 (0.77, 0.94) | Standardized Mortality Ratio (95%CI) |
|  |  | Sex and Age | M 80+ | 0.93 (0.84, 1.04) | Standardized Mortality Ratio (95%CI) |
|  |  | Sex and Age | F 50-64 | 0.86 (0.71, 1.05) | Standardized Mortality Ratio (95%CI) |
|  |  | Sex and Age | F 65-79 | 0.98 (0.86, 1.11) | Standardized Mortality Ratio (95%CI) |
|  |  | Sex and Age | F 80+ | 0.97 (0.88, 1.08) | Standardized Mortality Ratio (95%CI) |
| Covid-19 in Brazil in 2020: impact on deaths from cancer and cardiovascular diseases | Country, Brasil | Aggregated |  | 0.90 (0.90, 0.91) | Observed-to-expected ratio (95%CI) |
|  |  | Territory | North | 0.91 (0.89, 0.93) | Observed-to-expected ratio (95%CI) |
|  |  | Territory | Northeast | 0.90 (0.89, 0.91) | Observed-to-expected ratio (95%CI) |
|  |  | Territory | Southeast | 0.90 (0.90, 0.91) | Observed-to-expected ratio (95%CI) |
|  |  | Territory | South | 0.92 (0.91, 0.93) | Observed-to-expected ratio (95%CI) |
|  |  | Territory | Midwest | 0.89 (0.87, 0.91) | Observed-to-expected ratio (95%CI) |
| The US Midlife Mortality Crisis Continues: Excess Cause-Specific Mortality During 2020 | Country, USA | Sex | Males | 266 | Excess deaths |
|  |  | Sex | Females | -2429 | Excess deaths |
|  |  | Sex and Age | M <15 | -24 | Excess deaths |
|  |  | Sex and Age | M 15-24 | 9 | Excess deaths |
|  |  | Sex and Age | M 25-44 | 41 | Excess deaths |
|  |  | Sex and Age | M 45-64 | 672 | Excess deaths |
|  |  | Sex and Age | M 65 - 74 | 73 | Excess deaths |
|  |  | Sex and Age | M >=75 | -506 | Excess deaths |
|  |  | Sex and Age | F <15 | -17 | Excess deaths |
|  |  | Sex and Age | F 15-24 | -5 | Excess deaths |
|  |  | Sex and Age | F 25-44 | -330 | Excess deaths |
|  |  | Sex and Age | F 45-64 | -821 | Excess deaths |
|  |  | Sex and Age | F 65 - 74 | 675 | Excess deaths |
|  |  | Sex and Age | F >=75 | -1931 | Excess deaths |
| Child mortality in England during the first year of the COVID-19 pandemic | Country, UK | Aggregated |  | 1.02 (0.86, 1.21) | Observed-to-expected ratio |
| Excess natural-cause deaths in California by cause and setting: March 2020 through February 2021 | State, USA | Care setting | In hospital | −3,652 (−4,331, −2,968) | Excess deaths (95%CI) |
|  |  | Care setting | Out of hospital | 5,536 (5,090, 5,981) | Excess deaths (95%CI) |

**Table S4. Diabetes mellitus**

| **Study title** | **Territory** | **Level of aggregation** | **Specific aggregation** | **Expected vs observed** | **Unit of measure** |
| --- | --- | --- | --- | --- | --- |
| Excess mortality by specific causes of deaths in the city of São Paulo, Brazil, during the COVID-19 pandemic | City, San-Paolo, Brazil | Gender | Males | 1.1 (0.52, 1.85) | Standardized Mortality Ratio (95%CI) |
|  |  | Gender | Females | 1.1 (0.47, 2.19) | Standardized Mortality Ratio (95%CI) |
| Counting the Dead: COVID-19 and Mortality in Quebec and British Columbia During the First Wave | Territories, Canada | Territory | British Columbia | 29.1 | Excess deaths |
|  |  | Territory | Quebec | -48.1 | Excess deaths |
| Excess mortality in Wuhan city and other parts of China during the three months of the covid-19 outbreak: findings from nationwide mortality registries | Territories, China | City | Wuhan | 1.83 (1.08, 4.37) | Rate ratio (95%CI) |
|  |  | Territory | Hubei without Wuhan | 0.99 (0.72, 1.49) | Rate ratio (95%CI) |
|  |  | Territory | China without Hubei | 0.94 (0.84, 1.07) | Rate ratio (95%CI) |
| Temporal dynamic in the impact of COVID- 19 outbreak on cause-specific mortality in Guangzhou, China | City, China | Aggregated |  | 10.2 (-3.7, 22.0) | % variation (95%CI) |
|  |  | Gender | Males | 13.5 (-1.4, 25.6) | % variation (95%CI) |
|  |  | Gender | Females | 6.7 (-9.2, 19.0) | % variation (95%CI) |
|  |  | Age group | <25 | 120.4 (-31.6, 182.8) | % variation (95%CI) |
|  |  | Age group | 25-44 | 2.3 (-33.2, 26.6) | % variation (95%CI) |
|  |  | Age group | 45-64 | 4.5 (-13.1, 16.9) | % variation (95%CI) |
|  |  | Age group | 65-74 | 6.7 (-10.5, 19.4) | % variation (95%CI) |
|  |  | Age group | 75-84 | 4.3 (-12.0, 16.5) | % variation (95%CI) |
|  |  | Age group | 85+ | 37.6 (19.7, 52.0) | % variation (95%CI) |
|  |  | Setting of care | Hospital | 2.4 (-24.3, 21.6) | % variation (95%CI) |
|  |  | Setting of care | Outside hospitals | -19.1 (-46.1, -2.0) | % variation (95%CI) |
|  |  | Marital status | Unmarried | -21.9 (-51.2, -1.9) | % variation (95%CI) |
|  |  | Marital status | Married | -9.6 (-36.2, 6.9) | % variation (95%CI) |
|  |  | Marital status | Divorced | -66.9 (-119.3, -35.9) | % variation (95%CI) |
|  |  | Marital status | Widowed | 62.6 (21.9, 89.0) | % variation (95%CI) |
|  |  | Occupation class | Gold-collar | -24.5 (-84.8, 10.3) | % variation (95%CI) |
|  |  | Occupation class | White-collar | 7.7 (-39.4, 36.4) | % variation (95%CI) |
|  |  | Occupation class | Pink-collar | -38.7 (-73.9, -14.8) | % variation (95%CI) |
|  |  | Occupation class | Blue-collar | -1.7 (-25.5, 16.4) | % variation (95%CI) |
|  |  | Occupation class | Others | -16.6 (-43.5, 2.4) | % variation (95%CI) |
| Place and Underlying Cause of Death During the COVID-19 Pandemic: Retrospective Cohort Study of 3.5 Million Deaths in England and Wales, 2014 to 2020 | Countries, England and Wales | Aggregated |  | 683 (+32%) | Excess deaths (% variation) |
|  |  | Setting of care | Home | 296 (+52%) | Excess deaths (% variation) |
|  |  | Setting of care | Care home or hospice | 308 (+49%) | Excess deaths (% variation) |
|  |  | Setting of care | Hospital | 57 (+6%) | Excess deaths (% variation) |
| Variation in Cause-Specific Mortality Rates in Italy during the First Wave of the COVID-19 Pandemic: A Study Based on Nationwide Data | Country, Italy | Aggregated |  | 32.6% | % variation |
|  |  | Gender | Males | 35.8% | % variation |
|  |  | Gender | Females | 29.3% | % variation |
| Leading causes of excess mortality in Mexico during the COVID-19 pandemic 2020-2021: A death certificates study in a middle-income country | Country, Mexico | Aggregated |  | 80,294 (71,066, 89,522) | Excess deaths (95%CI) |
| Excess mortality associated with the COVID-19 pandemic in Latvia: a population-level analysis of all-cause and noncommunicable disease deaths in 2020 | Country, Latvia | Aggregated |  | 113 (-78, 353) | Excess deaths (95%CI) |
| Impact of the COVID-19 Pandemic on Total and Cause-Specific Mortality in Pavia, Northern Italy | Province, Italy | Sex | Males | 1.03 (0.82 ,1.30) | Standardized Mortality Ratio (95%CI) |
|  |  | Sex | Females | 1.13 (0.92, 1.40) | Standardized Mortality Ratio (95%CI) |
|  |  | Sex and Age | M 50-64 |  | Mortality Ratio (95%CI) |
|  |  | Sex and Age | M 65-79 |  | Mortality Ratio (95%CI) |
|  |  | Sex and Age | M 80+ |  | Mortality Ratio (95%CI) |
|  |  | Sex and Age | F 50-64 |  | Mortality Ratio (95%CI) |
|  |  | Sex and Age | F 65-79 |  | Mortality Ratio (95%CI) |
|  |  | Sex and Age | F 80+ |  | Mortality Ratio (95%CI) |
| The US Midlife Mortality Crisis Continues: Excess Cause-Specific Mortality During 2020 | Country, USA | Sex | Males | 7292 | Excess deaths |
|  |  | Sex | Females | 6433 | Excess deaths |
|  |  | Sex and Age | M <15 |  | Excess deaths |
|  |  | Sex and Age | M 15-24 |  | Excess deaths |
|  |  | Sex and Age | M 25-44 | 535 | Excess deaths |
|  |  | Sex and Age | M 45-64 | 2183 | Excess deaths |
|  |  | Sex and Age | M 65 - 74 | 1947 | Excess deaths |
|  |  | Sex and Age | M >=75 | 2626 | Excess deaths |
|  |  | Sex and Age | F <15 |  | Excess deaths |
|  |  | Sex and Age | F 15-24 |  | Excess deaths |
|  |  | Sex and Age | F 25-44 | 279 | Excess deaths |
|  |  | Sex and Age | F 45-64 | 1149 | Excess deaths |
|  |  | Sex and Age | F 65 - 74 | 1551 | Excess deaths |
|  |  | Sex and Age | F >=75 | 3455 | Excess deaths |
| Excess natural-cause deaths in California by cause and setting: March 2020 through February 2021 | State, USA | Care setting | In hospital | 51 (−143, 247) | Excess deaths (95%CI) |
|  |  | Care setting | Out of hospital | 2,050 (1,661, 2,443) | Excess deaths (95%CI) |

**Table S5. Suicides**

| **Study title** | **Territory** | **Level of aggregation** | **Specific aggregation** | **Expected vs observed** | **Unit of measure** |
| --- | --- | --- | --- | --- | --- |
| Counting the Dead: COVID-19 and Mortality in Quebec and British Columbia During the First Wave | Territories, Canada | Territory | British Columbia | -54.6 | Excess deaths |
|  |  | Territory | Quebec | -151.3 | Excess deaths |
| Excess mortality in Wuhan city and other parts of China during the three months of the covid-19 outbreak: findings from nationwide mortality registries | Territories, China | City | Wuhan | 1.66 (0.74, ∞) | Rate ratio (95%CI) |
|  |  | Territory | Hubei without Wuhan | 1.62 (1.15, 2.53) | Rate ratio (95%CI) |
|  |  | Territory | China without Hubei | 1.09 (0.93, 1.30) | Rate ratio (95%CI) |
| Temporal dynamic in the impact of COVID- 19 outbreak on cause-specific mortality in Guangzhou, China | City, China | Aggregated |  | 1.7 (-27.5, 21.1) | % variation (95%CI) |
|  |  | Gender | Males | 8.1 (-21.4, 27.5) | % variation (95%CI) |
|  |  | Gender | Females | -7.7 (-40.5, 12.0) | % variation (95%CI) |
|  |  | Age group | <25 | 3.3 (-37.9, 27.1) | % variation (95%CI) |
|  |  | Age group | 25-44 | -11.0 (-43.3, 10.0) | % variation (95%CI) |
|  |  | Age group | 45-64 | -5.1 (-37.1, 14.0) | % variation (95%CI) |
|  |  | Age group | 65-74 | 37.3 (0.5, 61.5) | % variation (95%CI) |
|  |  | Age group | 75-84 | 7.9 (-30.1, 33.6) | % variation (95%CI) |
|  |  | Age group | 85+ | 6.5 (-54.5, 40.7) | % variation (95%CI) |
|  |  | Setting of care | Hospital | 11.0 (-22.5, 31.2) | % variation (95%CI) |
|  |  | Setting of care | Outside hospitals | -1.7 (-31.9, 15.2) | % variation (95%CI) |
|  |  | Marital status | Unmarried | 2.9 (-31.4, 24.4) | % variation (95%CI) |
|  |  | Marital status | Married | 5.6 (-24.5, 24.4) | % variation (95%CI) |
|  |  | Marital status | Divorced | -33.6 (-78.5, - 8.1) | % variation (95%CI) |
|  |  | Marital status | Widowed | 19.7 (-27.3, 46.5) | % variation (95%CI) |
|  |  | Occupation class | Gold-collar | 30.4 (-28.4, 67.8) | % variation (95%CI) |
|  |  | Occupation class | White-collar | -10.7 (-58.8, 17.1) | % variation (95%CI) |
|  |  | Occupation class | Pink-collar | 41.2 (-7.7, 69.4) | % variation (95%CI) |
|  |  | Occupation class | Blue-collar | 18.1 (-17.9, 39.1) | % variation (95%CI) |
|  |  | Occupation class | Others | -9.3 (-38.9, 10.5) | % variation (95%CI) |
| Mortality From Drug Overdoses, Homicides, Unintentional Injuries, Motor Vehicle Crashes, and Suicides During the Pandemic, March-August 2020 | Country, USA | Aggregated |  | 0.91 (0.86, 0.96) | Observed-to-expected ratio (95%CI) |
|  |  | Time period | March | 0.94 (0.89, 0.98) | Observed-to-expected ratio (95%CI) |
|  |  | Time period | April | 0.84 (0.79, 0.89) | Observed-to-expected ratio (95%CI) |
|  |  | Time period | May | 0.87 (0.83, 0.92) | Observed-to-expected ratio (95%CI) |
|  |  | Time period | June | 0.93 (0.88, 0.98) | Observed-to-expected ratio (95%CI) |
|  |  | Time period | July | 0.94 (0.89, 1) | Observed-to-expected ratio (95%CI) |
|  |  | Time period | August | 0.91 (0.86, 0.96) | Observed-to-expected ratio (95%CI) |
| Variation in Cause-Specific Mortality Rates in Italy during the First Wave of the COVID-19 Pandemic: A Study Based on Nationwide Data | Country, Italy | Aggregated |  | -17.5% | % variation |
|  |  | Gender | Males | -13.2% | % variation |
|  |  | Gender | Females | -29.9% | % variation |
| Leading causes of excess mortality in Mexico during the COVID-19 pandemic 2020-2021: A death certificates study in a middle-income country | Country, Mexico | Aggregated |  | -211 (-2764, 2343) | Excess deaths (95%CI) |
| Impact of the COVID-19 Pandemic on Total and Cause-Specific Mortality in Pavia, Northern Italy | Province, Italy | Sex | Males | 0.91 (0.62, 1.33) | Standardized Mortality Ratio (95%CI) |
|  |  | Sex | Females | 1.0 (0.49, 2.03) | Standardized Mortality Ratio (95%CI) |
|  |  | Sex and Age | M 50-64 |  |  |
|  |  | Sex and Age | M 65-79 |  |  |
|  |  | Sex and Age | M 80+ |  |  |
|  |  | Sex and Age | F 50-64 |  |  |
|  |  | Sex and Age | F 65-79 |  |  |
|  |  | Sex and Age | F 80+ |  |  |
| Excess suicides in Brazil: Inequalities according to age groups and regions during the COVID-19 pandemic | Country, Brasil | Aggregated |  | 0.87 (0.77, 0.99) | Observed-to-expected ratio (95%CI) |
|  |  | Age | 10-29 | 0.81 (0.70, 1.23) | Observed-to-expected ratio (95%CI) |
|  |  | Age | 30-59 | 0.87 (0.79, 0.97) | Observed-to-expected ratio (95%CI) |
|  |  | Age | >=60 | 0.97 (0.83, 1.15) | Observed-to-expected ratio (95%CI) |
|  |  | Sex | Females | 0.85 (0.72, 1.04) | Observed-to-expected ratio (95%CI) |
|  |  | Sex and Age | 10-29 | 0.78 (0.65, 0.98) | Observed-to-expected ratio (95%CI) |
|  |  | Sex and Age | 30-59 | 0.88 (0.77, 1.03) | Observed-to-expected ratio (95%CI) |
|  |  | Sex and Age | >=60 | 0.93 (0.74, 1.26) | Observed-to-expected ratio (95%CI) |
|  |  | Sex | Males | 0.87 (0.79, 0.98) | Observed-to-expected ratio (95%CI) |
|  |  | Sex and Age | 10-29 | 0.81 (0.72, 0.93) | Observed-to-expected ratio (95%CI) |
|  |  | Sex and Age | 30-59 | 0.87 (0.80, 0.96) | Observed-to-expected ratio (95%CI) |
|  |  | Sex and Age | >=60 | 0.98 (0.86, 1.13) | Observed-to-expected ratio (95%CI) |
| Child mortality in England during the first year of the COVID-19 pandemic | Country, UK | Aggregated |  | 1.10 (0.85, 1.43) | Observed-to-expected ratio (95%CI) |

**Table S6. Cerebrovascular diseases**

| **Study title** | **Territory** | **Level of aggregation** | **Specific aggregation** | **Expected vs observed** | **Unit of measure** |
| --- | --- | --- | --- | --- | --- |
| Counting the Dead: COVID-19 and Mortality in Quebec and British Columbia During the First Wave | Territories, Canada | Territory | British Columbia | -53.6 | Excess deaths |
|  |  | Territory | Quebec | -127.1 | Excess deaths |
| Temporal dynamic in the impact of COVID- 19 outbreak on cause-specific mortality in Guangzhou, China | City, China | Aggregated |  | 0.3 (-4.5, 4.9) | % variation (95%CI) |
|  |  | Gender | Males | -3.2 (-8.5, 1.3) | % variation (95%CI) |
|  |  | Gender | Females | 4.3 (-0.9, 9.2) | % variation (95%CI) |
|  |  | Age group | <25 | -22.1 (-71.6, 12.8) | % variation (95%CI) |
|  |  | Age group | 25-44 | 13.2 (-0.6, 25.4) | % variation (95%CI) |
|  |  | Age group | 45-64 | -13.6 (-19.8, -7.5) | % variation (95%CI) |
|  |  | Age group | 65-74 | 5.0 (-0.9, 10.7) | % variation (95%CI) |
|  |  | Age group | 75-84 | -3.4 (-9.0, 1.8) | % variation (95%CI) |
|  |  | Age group | 85+ | 8.7 (3.1, 13.4) | % variation (95%CI) |
|  |  | Setting of care | Hospital | -2.3 (-7.8, 2.4) | % variation (95%CI) |
|  |  | Setting of care | Outside hospitals | 0.1 (-5.1, 4.5) | % variation (95%CI) |
|  |  | Marital status | Unmarried | 9.8 (-0.8, 18.8) | % variation (95%CI) |
|  |  | Marital status | Married | -3.6 (-9.0, 1.2) | % variation (95%CI) |
|  |  | Marital status | Divorced | 2.6 (-14.3, 8.1) | % variation (95%CI) |
|  |  | Marital status | Widowed | 3.7 (-1.3, 8.7) | % variation (95%CI) |
|  |  | Occupation class | Gold-collar | 6.4 (-8.9, 19.8) | % variation (95%CI) |
|  |  | Occupation class | White-collar | 4.1 (-17.2, 7.8) | % variation (95%CI) |
|  |  | Occupation class | Pink-collar | -12.2 (-26.3, -1.4) | % variation (95%CI) |
|  |  | Occupation class | Blue-collar | 1.1 (-4.2, 5.8) | % variation (95%CI) |
|  |  | Occupation class | Others | -2.0 (-7.2, 2.4) | % variation (95%CI) |
| Place and Underlying Cause of Death During the COVID-19 Pandemic: Retrospective Cohort Study of 3.5 Million Deaths in England and Wales, 2014 to 2020 | Countries, England and Wales | Aggregated |  | 971 (+11%) | Excess deaths (% variation) |
|  |  | Setting of care | Home | 530 (+51%) | Excess deaths (% variation) |
|  |  | Setting of care | Care home or hospice | 871 (+39%) | Excess deaths (% variation) |
|  |  | Setting of care | Hospital | -459 (-8%) | Excess deaths (% variation) |
| Excess Cerebrovascular Mortality in the United States During the COVID-19 Pandemic | States, USA | Aggregated |  | 918 | Excess deaths |
| Variation in Cause-Specific Mortality Rates in Italy during the First Wave of the COVID-19 Pandemic: A Study Based on Nationwide Data | Country, Italy | Aggregated |  | 5.8% | % variation |
|  |  | Gender | Males | 2.6% | % variation |
|  |  | Gender | Females | 7.4% | % variation |
| Leading causes of excess mortality in Mexico during the COVID-19 pandemic 2020-2021: A death certificates study in a middle-income country | Country, Mexico | Aggregated |  | 4626 (-531, 9782) | Excess deaths (95%CI) |
| Impact of the COVID-19 Pandemic on Total and Cause-Specific Mortality in Pavia, Northern Italy | Province, Italy | Sex | Males | 0.75 (0.64, 0.88) | Standardized Mortality Ratio (95%CI) |
|  |  | Sex | Females | 0.91 (0.82, 1.01) | Standardized Mortality Ratio (95%CI) |
|  |  | Sex and Age | M 50-64 |  |  |
|  |  | Sex and Age | M 65-79 |  |  |
|  |  | Sex and Age | M 80+ |  |  |
|  |  | Sex and Age | F 50-64 |  |  |
|  |  | Sex and Age | F 65-79 |  |  |
|  |  | Sex and Age | F 80+ |  |  |
| The US Midlife Mortality Crisis Continues: Excess Cause-Specific Mortality During 2020 | Country, USA | Sex | Males | 2881 | Excess deaths |
|  |  | Sex | Females | 2440 | Excess deaths |
|  |  | Sex and Age | M <15 |  | Excess deaths |
|  |  | Sex and Age | M 15-24 |  | Excess deaths |
|  |  | Sex and Age | M 25-44 | 187 | Excess deaths |
|  |  | Sex and Age | M 45-64 | 1121 | Excess deaths |
|  |  | Sex and Age | M 65 - 74 | 615 | Excess deaths |
|  |  | Sex and Age | M >=75 | 958 | Excess deaths |
|  |  | Sex and Age | F <15 |  | Excess deaths |
|  |  | Sex and Age | F 15-24 |  | Excess deaths |
|  |  | Sex and Age | F 25-44 | 74 | Excess deaths |
|  |  | Sex and Age | F 45-64 | 538 | Excess deaths |
|  |  | Sex and Age | F 65 - 74 | 694 | Excess deaths |
|  |  | Sex and Age | F >=75 | 1134 | Excess deaths |
| Excess natural-cause deaths in California by cause and setting: March 2020 through February 2021 | State, USA | Care setting | In hospital | −516 (−658, −373) | Excess deaths (95%CI) |
|  |  | Care setting | Out of hospital | 1,982 (1,450, 2,515) | Excess deaths (95%CI) |

**Table S7. Road accidents**

| **Study title** | **Territory** | **Level of aggregation** | **Specific aggregation** | **Expected vs observed** | **Unit of measure** |
| --- | --- | --- | --- | --- | --- |
| Excess mortality in Wuhan city and other parts of China during the three months of the covid-19 outbreak: findings from nationwide mortality registries | Territories, China | City | Wuhan | 0.63 (0.28, ∞) | Rate ratio (95%CI) |
|  |  | Territory | Hubei without Wuhan | 0.63 (0.44, 0.99) | Rate ratio (95%CI) |
|  |  | Territory | China without Hubei | 0.77 (0.68, 0.88) | Rate ratio (95%CI) |
| Temporal dynamic in the impact of COVID- 19 outbreak on cause-specific mortality in Guangzhou, China | City, China | Aggregated |  | -8.8 (- 33.2, 8.3) | % variation (95%CI) |
|  |  | Gender | Males | -11.6 (-36.6, 6.1) | % variation (95%CI) |
|  |  | Gender | Females | -1.7 (-29.8, 16.8) | % variation (95%CI) |
|  |  | Age group | <25 | -24.0 (-62.2, -2.6) | % variation (95%CI) |
|  |  | Age group | 25-44 | -9.5 (-39.2, 9.9) | % variation (95%CI) |
|  |  | Age group | 45-64 | -16.3 (-41.7, 2.0) | % variation (95%CI) |
|  |  | Age group | 65-74 | -1.8 (-30.0, 17.7) | % variation (95%CI) |
|  |  | Age group | 75-84 | 13.1 (-20.4, 33.8) | % variation (95%CI) |
|  |  | Age group | 85+ | 17.2 (-43.4, 51.2) | % variation (95%CI) |
|  |  | Setting of care | Hospital | 2.4 (-24.3, 21.6) | % variation (95%CI) |
|  |  | Setting of care | Outside hospitals | -19.1 (-46.1, -2.0) | % variation (95%CI) |
|  |  | Marital status | Unmarried | -21.9 (-51.2, -1.9) | % variation (95%CI) |
|  |  | Marital status | Married | -9.6 (-36.2, 6.9) | % variation (95%CI) |
|  |  | Marital status | Divorced | -66.9 (- 119.3, -35.9) | % variation (95%CI) |
|  |  | Marital status | Widowed | 62.6 (21.9, 89.0) | % variation (95%CI) |
|  |  | Occupation class | Gold-collar | -24.5 (-84.8, 10.3) | % variation (95%CI) |
|  |  | Occupation class | White-collar | 7.7 (-39.4, 36.4) | % variation (95%CI) |
|  |  | Occupation class | Pink-collar | -38.7 (-73.9, -14.8) | % variation (95%CI) |
|  |  | Occupation class | Blue-collar | -1.7 (-25.5, 16.4) | % variation (95%CI) |
|  |  | Occupation class | Others | -16.6 (-43.5, 2.4) | % variation (95%CI) |
| Mortality From Drug Overdoses, Homicides, Unintentional Injuries, Motor Vehicle Crashes, and Suicides During the Pandemic, March-August 2020 | Country, USA | Aggregated |  | 1.04 (0.95, 1.14) | Observed-to-expected ratio (95%CI) |
|  |  | Time period | March | 0.94 (0.86, 1.04) | Observed-to-expected ratio (95%CI) |
|  |  | Time period | April | 0.83 (0.76, 0.92) | Observed-to-expected ratio (95%CI) |
|  |  | Time period | May | 0.96 (0.89, 1.05) | Observed-to-expected ratio (95%CI) |
|  |  | Time period | June | 1.14 (1.05, 1.25) | Observed-to-expected ratio (95%CI) |
|  |  | Time period | July | 1.14 (1.05, 1.25) | Observed-to-expected ratio (95%CI) |
|  |  | Time period | August | 1.15 (1.06, 1.27) | Observed-to-expected ratio (95%CI) |
| Variation in Cause-Specific Mortality Rates in Italy during the First Wave of the COVID-19 Pandemic: A Study Based on Nationwide Data | Country, Italy | Aggregated |  | -58.5% | % variation |
|  |  | Gender | Males | -57.7% | % variation |
|  |  | Gender | Females | -60.5% | % variation |
| Leading causes of excess mortality in Mexico during the COVID-19 pandemic 2020-2021: A death certificates study in a middle-income country | Country, Mexico | Aggregated |  | 1837 (-314, 3989) | Excess deaths (95%CI) |
| Impact of the COVID-19 Pandemic on Total and Cause-Specific Mortality in Pavia, Northern Italy | Province, Italy | Sex | Males | 0.43 (0.23, 0.80) | Standardized Mortality Ratio (95%CI) |
|  |  | Sex | Females | 1.43 (0.68, 3.0) | Standardized Mortality Ratio (95%CI) |
|  |  | Sex and Age | M 50-64 |  |  |
|  |  | Sex and Age | M 65-79 |  |  |
|  |  | Sex and Age | M 80+ |  |  |
|  |  | Sex and Age | F 50-64 |  |  |
|  |  | Sex and Age | F 65-79 |  |  |
|  |  | Sex and Age | F 80+ |  |  |
| Child mortality in England during the first year of the COVID-19 pandemic | Country, UK | Aggregated |  | 1.15 (0.80, 1.65) | Observed-to-expected ratio (95%CI) |

**Table S8. Unintentional injuries**

| **Study title** | **Territory** | **Level of aggregation** | **Specific aggregation** | **Expected vs observed** | **Unit of measure** |
| --- | --- | --- | --- | --- | --- |
| Excess mortality in Wuhan city and other parts of China during the three months of the covid-19 outbreak: findings from nationwide mortality registries | Territories, China | City | Wuhan | 1.43 (0.81, 3.92) | Rate ratio (95%CI) |
|  |  | Territory | Hubei without Wuhan | 1.16 (0.83, 1.77) | Rate ratio (95%CI) |
|  |  | Territory | China without Hubei | 0.97 (0.87, 1.09) | Rate ratio (95%CI) |
| Mortality From Drug Overdoses, Homicides, Unintentional Injuries, Motor Vehicle Crashes, and Suicides During the Pandemic, March-August 2020 | Country, USA | Aggregated |  | 1.08 (1.01, 1.16) | Observed-to-expected ratio (95%CI) |
|  |  | Time period | March | 0.99 (0.94, 1.04) | Observed-to-expected ratio (95%CI) |
|  |  | Time period | April | 1.04 (0.97, 1.11) | Observed-to-expected ratio (95%CI) |
|  |  | Time period | May | 1.14 (1.06, 1.22) | Observed-to-expected ratio (95%CI) |
|  |  | Time period | June | 1.10 (1.03, 1.19) | Observed-to-expected ratio (95%CI) |
|  |  | Time period | July | 1.10 (1.02, 1.20) | Observed-to-expected ratio (95%CI) |
|  |  | Time period | August | 1.10 (1.01, 1.20) | Observed-to-expected ratio (95%CI) |
| Variation in Cause-Specific Mortality Rates in Italy during the First Wave of the COVID-19 Pandemic: A Study Based on Nationwide Data | Country, Italy | Aggregated |  | 25.3% | % variation |
|  |  | Gender | Males | 25.4% | % variation |
|  |  | Gender | Females | 26.1% | % variation |
| Impact of the COVID-19 Pandemic on Total and Cause-Specific Mortality in Pavia, Northern Italy | Province, Italy | Sex | Males | 1.30 (0.91, 1.85) | Standardized Mortality Ratio (95%CI) |
|  |  | Sex | Females | 0.65 (0.43, 1.01) | Standardized Mortality Ratio (95%CI) |
|  |  | Sex and Age | M 50-64 |  | Standardized Mortality Ratio (95%CI) |
|  |  | Sex and Age | M 65-79 |  | Standardized Mortality Ratio (95%CI) |
|  |  | Sex and Age | M 80+ |  | Standardized Mortality Ratio (95%CI) |
|  |  | Sex and Age | F 50-64 |  | Standardized Mortality Ratio (95%CI) |
|  |  | Sex and Age | F 65-79 |  | Standardized Mortality Ratio (95%CI) |
|  |  | Sex and Age | F 80+ |  | Standardized Mortality Ratio (95%CI) |

**Table S9. Respiratory system diseases**

| **Study title** | **Territory** | **Level of aggregation** | **Specific aggregation** | **Expected vs observed** | **Unit of measure** |
| --- | --- | --- | --- | --- | --- |
| Counting the Dead: COVID-19 and Mortality in Quebec and British Columbia During the First Wave | Territories, Canada | Territory | British Columbia | 20.5 | Excess deaths |
|  |  | Territory | Quebec | -213.5 | Excess deaths |
| Temporal dynamic in the impact of COVID- 19 outbreak on cause-specific mortality in Guangzhou, China | City, China | Aggregated |  | -37.4 (-42.6, -32.2) | % variation (95%CI) |
|  |  | Gender | Males | -37.5 (-42.9, -32.8) | % variation (95%CI) |
|  |  | Gender | Females | -37.1 (-42.9, -32.1) | % variation (95%CI) |
|  |  | Age group | <25 | -65.1 (-84.3, -49.4) | % variation (95%CI) |
|  |  | Age group | 25-44 | -29.6 (-49.1, -13.4) | % variation (95%CI) |
|  |  | Age group | 45-64 | -35.3 (-42.3, -28.2) | % variation (95%CI) |
|  |  | Age group | 65-74 | -33.3 (-40.1, -27.1) | % variation (95%CI) |
|  |  | Age group | 75-84 | -42.0 (-47.4, -36.7) | % variation (95%CI) |
|  |  | Age group | 85+ | -34.3 (-39.9, -29.1) | % variation (95%CI) |
|  |  | Setting of care | Hospital | -38.1 (-43.8, -33.0) | % variation (95%CI) |
|  |  | Setting of care | Outside hospitals | -38.1 (-43.7, -33.0) | % variation (95%CI) |
|  |  | Marital status | Unmarried | -42.6 (-52.5, -34.0) | % variation (95%CI) |
|  |  | Marital status | Married | -37.2 (-42.7, -32.2) | % variation (95%CI) |
|  |  | Marital status | Divorced | -42.2 (-55.1, -30.4) | % variation (95%CI) |
|  |  | Marital status | Widowed | -38.9 (-44.4, -33.8) | % variation (95%CI) |
|  |  | Occupation class | Gold-collar | -44.0 (-60.4, -29.7) | % variation (95%CI) |
|  |  | Occupation class | White-collar | -45.3 (-58.2, -33.5) | % variation (95%CI) |
|  |  | Occupation class | Pink-collar | -38.5 (-55.3, -24.5) | % variation (95%CI) |
|  |  | Occupation class | Blue-collar | -37.2 (-43.5, -32.0) | % variation (95%CI) |
|  |  | Occupation class | Others | -38.1 (-43.8, -33.3) | % variation (95%CI) |
| Effects of COVID-19 on mortality: A 5-year population-based study in Oman | Country, Oman | Aggregated |  | 9 (5, 12) | Observed-to-expected ratio (95%CI) |
|  |  | Care setting | Home |  |  |
|  |  | Care setting | In hospitals | 9 (7, 13) | Observed-to-expected ratio (95%CI) |
| Excess deaths from COVID-19 and other causes by region, neighbourhood deprivation level and place of death during the first 30 weeks of the pandemic in England and Wales: A retrospective registry study | Countries, England and Wales | Aggregated |  | -2710 (-3492, -1928) | Excess deaths (95%CI) |
|  |  | Gender | Males | -768 (-1063,-473) | Excess deaths (95%CI) |
|  |  | Gender | Females | -1942 (-2236,-1648) | Excess deaths (95%CI) |
|  |  | Age group | 0-14 | -14 (-16,-12) | Excess deaths (95%CI) |
|  |  | Age group | 15-44 | -11 (-17,-5) | Excess deaths (95%CI) |
|  |  | Age group | 45-65 | 17 (-19, 54) | Excess deaths (95%CI) |
|  |  | Age group | 65-74 | -397 (-476, -318) | Excess deaths (95%CI) |
|  |  | Age group | 75-84 | -1051 (-1200,-902) | Excess deaths (95%CI) |
|  |  | Age group | 85+ | -1959 (-2170,-1747) | Excess deaths (95%CI) |
|  |  | Territory | North East | -208 (-231,-186) | Excess deaths (95%CI) |
|  |  | Territory | North West | -559 (-616,-502) | Excess deaths (95%CI) |
|  |  | Territory | Yorkshire & Humber | -260 (-298, -222) | Excess deaths (95%CI) |
|  |  | Territory | East Midlands | -245 (-276,-214) | Excess deaths (95%CI) |
|  |  | Territory | West Midlands | -223 (-263,-182) | Excess deaths (95%CI) |
|  |  | Territory | East of England | -174 (-213,-135) | Excess deaths (95%CI) |
|  |  | Territory | London | 15 (-16, 47) | Excess deaths (95%CI) |
|  |  | Territory | South East Coast | -201 (-234,-169) | Excess deaths (95%CI) |
|  |  | Territory | South Central | -145 (-169,-121) | Excess deaths (95%CI) |
|  |  | Territory | South West | -263 (-299,-226) | Excess deaths (95%CI) |
|  |  | Territory | Wales | -285 (-312,-258) | Excess deaths (95%CI) |
|  |  | Deprivation quintiles | 1 (least deprived) | -617 (-688,-545) | Excess deaths (95%CI) |
|  |  | Deprivation quintiles | 2 | -587 (-669,-506) | Excess deaths (95%CI) |
|  |  | Deprivation quintiles | 3 | -456 (-541,-372) | Excess deaths (95%CI) |
|  |  | Deprivation quintiles | 4 | -581 (-670,-493) | Excess deaths (95%CI) |
|  |  | Deprivation quintiles | 5 (most deprived) | -351 (-448,-253) | Excess deaths (95%CI) |
|  |  | Setting of care | Care home | 879 (806,953) | Excess deaths (95%CI) |
|  |  | Setting of care | Home | 1598 (1501, 1694) | Excess deaths (95%CI) |
|  |  | Setting of care | Hospice | -86 (-96,-77) | Excess deaths (95%CI) |
|  |  | Setting of care | Hospital | -5256 (-5576,-4936) | Excess deaths (95%CI) |
|  |  | Setting of care | Other/ Unknown | 148 (140,155) | Excess deaths (95%CI) |
| Impact of the COVID-19 Pandemic on Total and Cause-Specific Mortality in Pavia, Northern Italy | Province, Italy | Sex | Males | 1.44 (1.28, 1.61) | Standardized Mortality Ratio (95%CI) |
|  |  | Sex | Females | 1.30 (1.16, 1.47) | Standardized Mortality Ratio (95%CI) |
|  |  | Sex and Age | M 50-64 |  |  |
|  |  | Sex and Age | M 65-79 |  |  |
|  |  | Sex and Age | M 80+ |  |  |
|  |  | Sex and Age | F 50-64 |  |  |
|  |  | Sex and Age | F 65-79 |  |  |
|  |  | Sex and Age | F 80+ |  |  |

**Table S10. Chronic respiratory diseases**

| **Study title** | **Territory** | **Level of aggregation** | **Specific aggregation** | **Expected vs observed** | **Unit of measure** |
| --- | --- | --- | --- | --- | --- |
| Excess mortality in Wuhan city and other parts of China during the three months of the covid-19 outbreak: findings from nationwide mortality registries | Territories, China | City | Wuhan | 1.13 (0.73, 2.15) | Rate ratio (95%CI) |
|  |  | Territory | Hubei without Wuhan | 0.75 (0.58, 1.01) | Rate ratio (95%CI) |
|  |  | Territory | China without Hubei | 0.82 (0.71, 0.96) | Rate ratio (95%CI) |
| Temporal dynamic in the impact of COVID- 19 outbreak on cause-specific mortality in Guangzhou, China | City, China | Aggregated |  | -22.1 (-30.6, -14.4) | % variation (95%CI) |
|  |  | Gender | Males | -21.4 (-30.3, -13.9) | % variation (95%CI) |
|  |  | Gender | Females | -23.9 (-33.4, -15.4) | % variation (95%CI) |
|  |  | Age group | <25 | -100.0 (- 268.9, -36.5) | % variation (95%CI) |
|  |  | Age group | 25-44 | -28.0 (-90.3, 10.0) | % variation (95%CI) |
|  |  | Age group | 45-64 | -13.6 (-28.8, -2.4) | % variation (95%CI) |
|  |  | Age group | 65-74 | -14.4 (-25.2, -4.7) | % variation (95%CI) |
|  |  | Age group | 75-84 | -31.6 (-40.7, -23.5) | % variation (95%CI) |
|  |  | Age group | 85+ | -15.7 (-25.8, -7.6) | % variation (95%CI) |
|  |  | Setting of care | Hospital | -30.6 (-39.7, -22.2) | % variation (95%CI) |
|  |  | Setting of care | Outside hospitals | -17.4 (-26.1, -10.1) | % variation (95%CI) |
|  |  | Marital status | Unmarried | -20.1 (-38.0, -5.3) | % variation (95%CI) |
|  |  | Marital status | Married | -23.0 (-31.5, -15.6) | % variation (95%CI) |
|  |  | Marital status | Divorced | -16.4 (-42.7, 2.5) | % variation (95%CI) |
|  |  | Marital status | Widowed | -23.5 (-33.3, -15.4) | % variation (95%CI) |
|  |  | Occupation class | Gold-collar | -19.4 (-51.2, 6.4) | % variation (95%CI) |
|  |  | Occupation class | White-collar | -24.0 (-56.1, -3.2) | % variation (95%CI) |
|  |  | Occupation class | Pink-collar | -41.5 (-68.0, -21.2) | % variation (95%CI) |
|  |  | Occupation class | Blue-collar | -20.8 (-29.7, -13.1) | % variation (95%CI) |
|  |  | Occupation class | Others | -24.1 (-32.8, -16.7) | % variation (95%CI) |
| Variation in Cause-Specific Mortality Rates in Italy during the First Wave of the COVID-19 Pandemic: A Study Based on Nationwide Data | Country, Italy | Aggregated |  | 18.7% | % variation |
|  |  | Gender | Males | 16.5% | % variation |
|  |  | Gender | Females | 18.8% | % variation |
| Leading causes of excess mortality in Mexico during the COVID-19 pandemic 2020-2021: A death certificates study in a middle-income country | Country, Mexico | Aggregated |  | -3859 (-9178, 1460) | Excess deaths (95%CI) |
| Excess mortality associated with the COVID-19 pandemic in Latvia: a population-level analysis of all-cause and noncommunicable disease deaths in 2020 | Country, Latvia | Aggregated |  | 14 (-174, 154) | Excess deaths (95%CI) |
| Impact of the COVID-19 Pandemic on Total and Cause-Specific Mortality in Pavia, Northern Italy | Province, Italy | Sex | Males | 1.17 (0.96, 1.44) | Standardized Mortality Ratio (95%CI) |
|  |  | Sex | Females | 1.09 (0.87, 1.36) | Standardized Mortality Ratio (95%CI) |
|  |  | Sex and Age | M 50-64 |  |  |
|  |  | Sex and Age | M 65-79 |  |  |
|  |  | Sex and Age | M 80+ |  |  |
|  |  | Sex and Age | F 50-64 |  |  |
|  |  | Sex and Age | F 65-79 |  |  |
|  |  | Sex and Age | F 80+ |  |  |

**Table S11. Hypertensive disorders**

| **Study title** | **Territory** | **Level of aggregation** | **Specific aggregation** | **Expected vs observed** | **Unit of measure** |
| --- | --- | --- | --- | --- | --- |
| Excess mortality in Wuhan city and other parts of China during the three months of the covid-19 outbreak: findings from nationwide mortality registries | Territories, China | City | Wuhan | 2.00 (1.24, 4.25) | Rate ratio (95%CI) |
|  |  | Territory | Hubei without Wuhan | 0.82 (0.63, 1.13) | Rate ratio (95%CI) |
|  |  | Territory | China without Hubei | 0.77 (0.60, 1.04) | Rate ratio (95%CI) |
| Variation in Cause-Specific Mortality Rates in Italy during the First Wave of the COVID-19 Pandemic: A Study Based on Nationwide Data | Country, Italy | Aggregated |  | 28.5% | % variation |
|  |  | Gender | Males | 30.0% | % variation |
|  |  | Gender | Females | 27.8% | % variation |
| Leading causes of excess mortality in Mexico during the COVID-19 pandemic 2020-2021: A death certificates study in a middle-income country | Country, Mexico | Aggregated |  | 13,430 (8813, 18,047) | Excess deaths (95%CI) |

**Table S12. Hischemic heart diseases**

| **Study title** | **Territory** | **Level of aggregation** | **Specific aggregation** | **Expected vs observed** | **Unit of measure** |
| --- | --- | --- | --- | --- | --- |
| Excess mortality in Wuhan city and other parts of China during the three months of the covid-19 outbreak: findings from nationwide mortality registries | Territories, China | City | Wuhan | 1.24 (0.83, 2.19) | Rate ratio (95%CI) |
|  |  | Territory | Hubei without Wuhan | 1.03 (0.84, 1.30) | Rate ratio (95%CI) |
|  |  | Territory | China without Hubei | 0.96 (0.86, 1.07) | Rate ratio (95%CI) |
| Variation in Cause-Specific Mortality Rates in Italy during the First Wave of the COVID-19 Pandemic: A Study Based on Nationwide Data | Country, Italy | Aggregated |  | -0.9% | % variation |
|  |  | Gender | Males | 1.1% | % variation |
|  |  | Gender | Females | -3.5% | % variation |
| Leading causes of excess mortality in Mexico during the COVID-19 pandemic 2020-2021: A death certificates study in a middle-income country | Country, Mexico | Aggregated |  | 81,136 (70,765, 91,506) | Excess deaths (95%CI) |
| Impact of the COVID-19 Pandemic on Total and Cause-Specific Mortality in Pavia, Northern Italy | Province, Italy | Sex | Males | 0.91 (0.81, 1.03) | Standardized Mortality Ratio (95%CI) |
|  |  | Sex | Females | 0.87 (0.77, 0.99) | Standardized Mortality Ratio (95%CI) |
|  |  | Sex and Age | M 50-64 |  |  |
|  |  | Sex and Age | M 65-79 |  |  |
|  |  | Sex and Age | M 80+ |  |  |
|  |  | Sex and Age | F 50-64 |  |  |
|  |  | Sex and Age | F 65-79 |  |  |
|  |  | Sex and Age | F 80+ |  |  |

**Table S13. Kidney diseases**

| **Study title** | **Territory** | **Level of aggregation** | **Specific aggregation** | **Expected vs observed** | **Unit of measure** |
| --- | --- | --- | --- | --- | --- |
| Counting the Dead: COVID-19 and Mortality in Quebec and British Columbia During the First Wave | Territories, Canada | Territory | British Columbia | 9.3 | Excess deaths |
|  |  | Territory | Quebec | -46.6 | Excess deaths |
| Excess mortality in Wuhan city and other parts of China during the three months of the covid-19 outbreak: findings from nationwide mortality registries | Territories, China | City | Wuhan | 1.51 (0.60, ∞) | Rate ratio (95%CI) |
|  |  | Territory | Hubei without Wuhan | 1.10 (0.70, 2.16) | Rate ratio (95%CI) |
|  |  | Territory | China without Hubei | 0.89 (0.76, 1.07) | Rate ratio (95%CI) |
| Leading causes of excess mortality in Mexico during the COVID-19 pandemic 2020-2021: A death certificates study in a middle-income country | Country, Mexico | Aggregated |  | 681 (-3329, 4691) | Excess deaths (95%CI) |

**Table S14. Alzheimer disease**

| **Study title** | **Territory** | **Level of aggregation** | **Specific aggregation** | **Expected vs observed** | **Unit of measure** |
| --- | --- | --- | --- | --- | --- |
| Counting the Dead: COVID-19 and Mortality in Quebec and British Columbia During the First Wave | Territories, Canada | Territory | British Columbia | 29.3 | Excess deaths |
|  |  | Territory | Quebec | -38.5 | Excess deaths |
| Excess Cerebrovascular Mortality in the United States During the COVID-19 Pandemic | States, USA | Aggregated |  | 2910 | Excess deaths |
| The US Midlife Mortality Crisis Continues: Excess Cause-Specific Mortality During 2020 | Country, USA | Sex | Males | 2046 | Excess deaths |
|  |  | Sex | Females | 7716 | Excess deaths |
|  |  | Sex and Age | M <15 |  | Excess deaths |
|  |  | Sex and Age | M 15-24 |  | Excess deaths |
|  |  | Sex and Age | M 25-44 |  | Excess deaths |
|  |  | Sex and Age | M 45-64 | 24 | Excess deaths |
|  |  | Sex and Age | M 65 - 74 | 247 | Excess deaths |
|  |  | Sex and Age | M >=75 | 1775 | Excess deaths |
|  |  | Sex and Age | F <15 |  | Excess deaths |
|  |  | Sex and Age | F 15-24 |  | Excess deaths |
|  |  | Sex and Age | F 25-44 |  | Excess deaths |
|  |  | Sex and Age | F 45-64 | 14 | Excess deaths |
|  |  | Sex and Age | F 65 - 74 | 685 | Excess deaths |
|  |  | Sex and Age | F >=75 | 7018 | Excess deaths |
| Excess natural-cause deaths in California by cause and setting: March 2020 through February 2021 | State, USA | Care setting | In hospital | −346 (−624, −69) | Excess deaths (95%CI) |
|  |  | Care setting | Out of hospital | 2,587 (1,657, 3,515) | Excess deaths (95%CI) |

**Table S15. Dementia and Alzheimer disease**

| **Study title** | **Territory** | **Level of aggregation** | **Specific aggregation** | **Expected vs observed** | **Unit of measure** |
| --- | --- | --- | --- | --- | --- |
| Place and Underlying Cause of Death During the COVID-19 Pandemic: Retrospective Cohort Study of 3.5 Million Deaths in England and Wales, 2014 to 2020 | Countries, England and Wales | Aggregated |  | 5933 (+28%) | Excess deaths (% variation) |
|  |  | Setting of care | Home | 1096 (+45%) | Excess deaths (% variation) |
|  |  | Setting of care | Care home or hospice | 6267 (+45%) | Excess deaths (% variation) |
|  |  | Setting of care | Hospital | -1500 (-31%) | Excess deaths (% variation) |
| Variation in Cause-Specific Mortality Rates in Italy during the First Wave of the COVID-19 Pandemic: A Study Based on Nationwide Data | Country, Italy | Aggregated |  | 37.4% | % variation |
|  |  | Gender | Males | 30.3% | % variation |
|  |  | Gender | Females | 40.4% | % variation |
| Impact of the COVID-19 Pandemic on Total and Cause-Specific Mortality in Pavia, Northern Italy | Province, Italy | Sex | Males | 1.24 (1.04, 1.49) | Standardized Mortality Ratio (95%CI) |
|  |  | Sex | Females | 1.10 (0.97, 1.24) | Standardized Mortality Ratio (95%CI) |
|  |  | Sex and Age | M 50-64 |  |  |
|  |  | Sex and Age | M 65-79 |  |  |
|  |  | Sex and Age | M 80+ |  |  |
|  |  | Sex and Age | F 50-64 |  |  |
|  |  | Sex and Age | F 65-79 |  |  |
|  |  | Sex and Age | F 80+ |  |  |

**Table S16. Drugs overdose**

| **Study title** | **Territory** | **Level of aggregation** | **Specific aggregation** | **Expected vs observed** | **Unit of measure** |
| --- | --- | --- | --- | --- | --- |
| Mortality From Drug Overdoses, Homicides, Unintentional Injuries, Motor Vehicle Crashes, and Suicides During the Pandemic, March-August 2020 | Country, USA | Aggregated |  | 1.28 (1.15, 1.44) | Observed-to-expected ratio (95%CI) |
|  |  | Time period | March | 1.13 (1.04, 1.23) | Observed-to-expected ratio (95%CI) |
|  |  | Time period | April | 1.23 (1.13, 1.36) | Observed-to-expected ratio (95%CI) |
|  |  | Time period | May | 1.47 (1.32, 1.65) | Observed-to-expected ratio (95%CI) |
|  |  | Time period | June | 1.27 (1.13, 1.45) | Observed-to-expected ratio (95%CI) |
|  |  | Time period | July | 1.31 (1.15, 1.51) | Observed-to-expected ratio (95%CI) |
|  |  | Time period | August | 1.25 (1.1, 1.46) | Observed-to-expected ratio (95%CI) |
| Child mortality in England during the first year of the COVID-19 pandemic | Country, UK | Aggregated |  | 0.50 (0.23, 1.07) | Observed-to-expected ratio (95%CI) |

**Table S17. Homicides**

| **Study title** | **Territory** | **Level of aggregation** | **Specific aggregation** | **Expected vs observed** | **Unit of measure** |
| --- | --- | --- | --- | --- | --- |
| Mortality From Drug Overdoses, Homicides, Unintentional Injuries, Motor Vehicle Crashes, and Suicides During the Pandemic, March-August 2020 | Country, USA | Aggregated |  | 1.19 (1.1, 1.31) | Observed-to-expected ratio (95%CI) |
|  |  | Time period | March | 1.06 (0.98, 1.16) | Observed-to-expected ratio (95%CI) |
|  |  | Time period | April | 1.09 (1.01, 1.19) | Observed-to-expected ratio (95%CI) |
|  |  | Time period | May | 1.17 (1.08, 1.28) | Observed-to-expected ratio (95%CI) |
|  |  | Time period | June | 1.25 (1.14, 1.37) | Observed-to-expected ratio (95%CI) |
|  |  | Time period | July | 1.27 (1.17, 1.40) | Observed-to-expected ratio (95%CI) |
|  |  | Time period | August | 1.29 (1.17, 1.44) | Observed-to-expected ratio (95%CI) |
| Leading causes of excess mortality in Mexico during the COVID-19 pandemic 2020-2021: A death certificates study in a middle-income country | Country, Mexico | Aggregated |  | -737 (-5321, 3847) | Excess deaths (95%CI) |

**Table S18. Cardiovascuar diseases and diabetes mellitus**

| **Study title** | **Territory** | **Level of aggregation** | **Specific aggregation** | **Expected vs observed** | **Unit of measure** |
| --- | --- | --- | --- | --- | --- |
| Excess deaths from COVID-19 and other causes by region, neighbourhood deprivation level and place of death during the first 30 weeks of the pandemic in England and Wales: A retrospective registry study | Countries, England and Wales | Aggregated |  | 6887 (5999, 7774) | Excess deaths (95%CI) |
|  |  | Gender | Males | 3486 (3125, 3847) | Excess deaths (95%CI) |
|  |  | Gender | Females | 3388 (3075, 3702) | Excess deaths (95%CI) |
|  |  | Age group | 0-14 | 4 (1, 6) | Excess deaths (95%CI) |
|  |  | Age group | 15-44 | 237 (224, 249) | Excess deaths (95%CI) |
|  |  | Age group | 45-65 | 1379 (1314, 1444) | Excess deaths (95%CI) |
|  |  | Age group | 65-74 | 1050 (965, 1136) | Excess deaths (95%CI) |
|  |  | Age group | 75-84 | 1359 (1199, 1520) | Excess deaths (95%CI) |
|  |  | Age group | 85+ | 1907 (1673, 2142) | Excess deaths (95%CI) |
|  |  | Territory | North East | 316 (291, 341) | Excess deaths (95%CI) |
|  |  | Territory | North West | 488 (427, 549) | Excess deaths (95%CI) |
|  |  | Territory | Yorkshire & Humber | 469 (423, 516) | Excess deaths (95%CI) |
|  |  | Territory | East Midlands | 733 (692, 773) | Excess deaths (95%CI) |
|  |  | Territory | West Midlands | 1106 (1055, 1156) | Excess deaths (95%CI) |
|  |  | Territory | East of England | 463 (408, 518) | Excess deaths (95%CI) |
|  |  | Territory | London | 855 (809, 902) | Excess deaths (95%CI) |
|  |  | Territory | South East Coast | 774 (734, 815) | Excess deaths (95%CI) |
|  |  | Territory | South Central | 648 (616, 680) | Excess deaths (95%CI) |
|  |  | Territory | South West | 846 (794, 898) | Excess deaths (95%CI) |
|  |  | Territory | Wales | 367 (333, 400) | Excess deaths (95%CI) |
|  |  | Deprivation quintiles | 1 (least deprived) | 1227 (1131, 1323) | Excess deaths (95%CI) |
|  |  | Deprivation quintiles | 2 | 1137 (1034, 1241) | Excess deaths (95%CI) |
|  |  | Deprivation quintiles | 3 | 1746 (1640, 1851) | Excess deaths (95%CI) |
|  |  | Deprivation quintiles | 4 | 1301 (1197, 1405) | Excess deaths (95%CI) |
|  |  | Deprivation quintiles | 5 (most deprived) | 1668 (1566, 1770) | Excess deaths (95%CI) |
|  |  | Setting of care | Care home | 2736 (2635, 2836) | Excess deaths (95%CI) |
|  |  | Setting of care | Home | 6667 (6481, 6852) | Excess deaths (95%CI) |
|  |  | Setting of care | Hospice | -3 (-14, 8) | Excess deaths (95%CI) |
|  |  | Setting of care | Hospital | -3082 (-3379,-2786) | Excess deaths (95%CI) |
|  |  | Setting of care | Other/ Unknown | 249 (225, 272) | Excess deaths (95%CI) |

**Table S19. Infectious diseases (excluding COVID-19)**

| **Study title** | **Territory** | **Level of aggregation** | **Specific aggregation** | **Expected vs observed** | **Unit of measure** |
| --- | --- | --- | --- | --- | --- |
| Variation in Cause-Specific Mortality Rates in Italy during the First Wave of the COVID-19 Pandemic: A Study Based on Nationwide Data | Country, Italy | Aggregated |  | -5.7% | % variation |
|  |  | Gender | Males | -3.3% | % variation |
|  |  | Gender | Females | -9.0% | % variation |
| Leading causes of excess mortality in Mexico during the COVID-19 pandemic 2020-2021: A death certificates study in a middle-income country | Country, Mexico | Aggregated |  | -9992 (-14,718 to -5267) | Excess deaths (95%CI) |
| Impact of the COVID-19 Pandemic on Total and Cause-Specific Mortality in Pavia, Northern Italy | Province, Italy | Sex | Males | 1.30 (1.06, 1.59) | Standardized Mortality Ratio (95%CI) |
|  |  | Sex | Females | 1.31 (1.08, 1.58) | Standardized Mortality Ratio (95%CI) |
|  |  | Sex and Age | M 50-64 |  |  |
|  |  | Sex and Age | M 65-79 |  |  |
|  |  | Sex and Age | M 80+ |  |  |
|  |  | Sex and Age | F 50-64 |  |  |
|  |  | Sex and Age | F 65-79 |  |  |
|  |  | Sex and Age | F 80+ |  |  |
| Child mortality in England during the first year of the COVID-19 pandemic | Country, UK | Aggregated |  | 0.49 (0.38, 0.64) | Observed-to-expected ratio (95%CI) |
| Excess natural-cause deaths in California by cause and setting: March 2020 through February 2021 | State, USA | Care setting | In hospital | −134 (−404, 139) | Excess deaths (95%CI) |
|  |  | Care setting | Out of hospital | 178 (59, 298) | Excess deaths (95%CI) |

**Table S20. Parkinson disease**

| **Study title** | **Territory** | **Level of aggregation** | **Specific aggregation** | **Expected vs observed** | **Unit of measure** |
| --- | --- | --- | --- | --- | --- |
| Place and Underlying Cause of Death During the COVID-19 Pandemic: Retrospective Cohort Study of 3.5 Million Deaths in England and Wales, 2014 to 2020 | Countries, England and Wales | Aggregated |  | 544 (+26%) | Excess deaths (% variation) |
|  |  | Setting of care | Home | 210 (+60%) | Excess deaths (% variation) |
|  |  | Setting of care | Care home or hospice | 357 (+34%) | Excess deaths (% variation) |
|  |  | Setting of care | Hospital | -23 (-3%) | Excess deaths (% variation) |

**Table S21. Liver diseases**

| **Study title** | **Territory** | **Level of aggregation** | **Specific aggregation** | **Expected vs observed** | **Unit of measure** |
| --- | --- | --- | --- | --- | --- |
| Place and Underlying Cause of Death During the COVID-19 Pandemic: Retrospective Cohort Study of 3.5 Million Deaths in England and Wales, 2014 to 2020 | Countries, England and Wales | Aggregated |  | 213 (+8%) | Excess deaths (% variation) |
|  |  | Setting of care | Home | 188 (+31%) | Excess deaths (% variation) |
|  |  | Setting of care | Care home or hospice | 26 (+16%) | Excess deaths (% variation) |
|  |  | Setting of care | Hospital | 9 (0%) | Excess deaths (% variation) |

**Table S22. Endocrinuous, nutritional and metabolic diseases.**

| **Study title** | **Territory** | **Level of aggregation** | **Specific aggregation** | **Expected vs observed** | **Unit of measure** |
| --- | --- | --- | --- | --- | --- |
| Impact of the COVID-19 Pandemic on Total and Cause-Specific Mortality in Pavia, Northern Italy | Province, Italy | Sex | Males | 1.09 (0.89, 1.33) | Standardized Mortality Ratio (95%CI) |
|  |  | Sex | Females | 1.10 (0.92, 1.31) | Standardized Mortality Ratio (95%CI) |
|  |  | Sex and Age | M 50-64 |  |  |
|  |  | Sex and Age | M 65-79 |  |  |
|  |  | Sex and Age | M 80+ |  |  |
|  |  | Sex and Age | F 50-64 |  |  |
|  |  | Sex and Age | F 65-79 |  |  |
|  |  | Sex and Age | F 80+ |  |  |
| Excess natural-cause deaths in California by cause and setting: March 2020 through February 2021 | State, USA | Care setting | In hospital | −22 (−203, 160) | Excess deaths (95%CI) |
|  |  | Care setting | Out of hospital | 919 (688, 1,152) | Excess deaths (95%CI) |

**Table S23. Digestive system diseases**

| **Study title** | **Territory** | **Level of aggregation** | **Specific aggregation** | **Expected vs observed** | **Unit of measure** |
| --- | --- | --- | --- | --- | --- |
| Leading causes of excess mortality in Mexico during the COVID-19 pandemic 2020-2021: A death certificates study in a middle-income country | Country, Mexico | Aggregated |  | -7025 (-14,990, 939) | Excess deaths (95%CI) |
| Impact of the COVID-19 Pandemic on Total and Cause-Specific Mortality in Pavia, Northern Italy | Province, Italy | Sex | Males | 0.93 (0.75, 1.14) | Standardized Mortality Ratio (95%CI) |
|  |  | Sex | Females | 0.86 (0.72, 1.05) | Standardized Mortality Ratio (95%CI) |
|  |  | Sex and Age | M 50-64 |  |  |
|  |  | Sex and Age | M 65-79 |  |  |
|  |  | Sex and Age | M 80+ |  |  |
|  |  | Sex and Age | F 50-64 |  |  |
|  |  | Sex and Age | F 65-79 |  |  |
|  |  | Sex and Age | F 80+ |  |  |
| Excess natural-cause deaths in California by cause and setting: March 2020 through February 2021 | State, USA | Care setting | In hospital | 1 (−643, 650) | Excess deaths (95%CI) |
|  |  | Care setting | Out of hospital | 1,023 (955, 1,092) | Excess deaths (95%CI) |

**Table S24. Diseases of the nervous system and sense organs.**

| **Study title** | **Territory** | **Level of aggregation** | **Specific aggregation** | **Expected vs observed** | **Unit of measure** |
| --- | --- | --- | --- | --- | --- |
| Impact of the COVID-19 Pandemic on Total and Cause-Specific Mortality in Pavia, Northern Italy | Province, Italy | Sex | Males | 0.92 (0.76, 1.12) | Standardized Mortality Ratio (95%CI) |
|  |  | Sex | Females | 1.14 (0.98, 1.32) | Standardized Mortality Ratio (95%CI) |
|  |  | Sex and Age | M 50-64 |  |  |
|  |  | Sex and Age | M 65-79 |  |  |
|  |  | Sex and Age | M 80+ |  |  |
|  |  | Sex and Age | F 50-64 |  |  |
|  |  | Sex and Age | F 65-79 |  |  |
|  |  | Sex and Age | F 80+ |  |  |
| Excess natural-cause deaths in California by cause and setting: March 2020 through February 2021 | State, USA | Care setting | In hospital | −141 (−336, 57) | Excess deaths (95%CI) |
|  |  | Care setting | Out of hospital | 678 (253, 1,107) | Excess deaths (95%CI) |

**Table S25. Diseases of the genitourinary system.**

| **Study title** | **Territory** | **Level of aggregation** | **Specific aggregation** | **Expected vs observed** | **Unit of measure** |
| --- | --- | --- | --- | --- | --- |
| Impact of the COVID-19 Pandemic on Total and Cause-Specific Mortality in Pavia, Northern Italy | Province, Italy | Sex | Males | 0.81 (0.62, 1.06) | Standardized Mortality Ratio (95%CI) |
|  |  | Sex | Females | 1.02 (0.81, 1.28) | Standardized Mortality Ratio (95%CI) |
|  |  | Sex and Age | M 50-64 |  |  |
|  |  | Sex and Age | M 65-79 |  |  |
|  |  | Sex and Age | M 80+ |  |  |
|  |  | Sex and Age | F 50-64 |  |  |
|  |  | Sex and Age | F 65-79 |  |  |
|  |  | Sex and Age | F 80+ |  |  |
| Excess natural-cause deaths in California by cause and setting: March 2020 through February 2021 | State, USA | Care setting | In hospital | 89 (−164, 346) | Excess deaths (95%CI) |
|  |  | Care setting | Out of hospital | 472 (289, 658) | Excess deaths (95%CI) |

**Table S26. Influenza and pneumonia (excluding COVID-19).**

| **Study title** | **Territory** | **Level of aggregation** | **Specific aggregation** | **Expected vs observed** | **Unit of measure** |
| --- | --- | --- | --- | --- | --- |
| Counting the Dead: COVID-19 and Mortality in Quebec and British Columbia During the First Wave | Territories, Canada | Territory | British Columbia | − 0.8 | Excess deaths |
|  |  | Territory | Quebec | − 100.4 | Excess deaths |
| Temporal dynamic in the impact of COVID- 19 outbreak on cause-specific mortality in Guangzhou, China | City, China | Aggregated |  | −49.2 (− 56.7, − 42.6) | % variation (95%CI) |
|  |  | Gender | Males | -50.7 (-58.2, - 44.7) | % variation (95%CI) |
|  |  | Gender | Females | -47.3 (-55.2, - 40.8) | % variation (95%CI) |
|  |  | Age group | <25 | -71.7 (-92.8, - 53.7) | % variation (95%CI) |
|  |  | Age group | 25-44 | -30.8 (-54.6, - 11.1) | % variation (95%CI) |
|  |  | Age group | 45-64 | -49.6 (-60.2, - 40.1) | % variation (95%CI) |
|  |  | Age group | 65-74 | -46.8 (-56.4, - 38.8) | % variation (95%CI) |
|  |  | Age group | 75-84 | -50.1 (-58.8, - 43.2) | % variation (95%CI) |
|  |  | Age group | 85+ | -48.8 (-56.8, - 41.8) | % variation (95%CI) |
|  |  | Setting of care | Hospital | -47.4 (-55.0, -41.3) | % variation (95%CI) |
|  |  | Setting of care | Outside hospitals | -57.8 (-66.0, -51.2) | % variation (95%CI) |
|  |  | Marital status | Unmarried | -55.8 (-68.2, -44.7) | % variation (95%CI) |
|  |  | Marital status | Married | -48.0 (-55.7, -41.3) | % variation (95%CI) |
|  |  | Marital status | Divorced | -55.8 (-73.7, -41.2) | % variation (95%CI) |
|  |  | Marital status | Widowed | -51.5 (-59.4, -44.1) | % variation (95%CI) |
|  |  | Occupation class | Gold-collar | -58.2 (-78.5, -40.8) | % variation (95%CI) |
|  |  | Occupation class | White-collar | -58.0 (-78.8, -41.9) | % variation (95%CI) |
|  |  | Occupation class | Pink-collar | -37.4 (-62.2, -16.5) | % variation (95%CI) |
|  |  | Occupation class | Blue-collar | -46.7 (-55.7, -38.1) | % variation (95%CI) |
|  |  | Occupation class | Others | -50.2 (-57.7, -43.7) | % variation (95%CI) |
| The US Midlife Mortality Crisis Continues: Excess Cause-Specific Mortality During 2020 | Country, USA | Sex | Males | 2441 | Excess deaths |
|  |  | Sex | Females | -54 | Excess deaths |
|  |  | Sex and Age | M <15 |  | Excess deaths |
|  |  | Sex and Age | M 15-24 |  | Excess deaths |
|  |  | Sex and Age | M 25-44 | 64 | Excess deaths |
|  |  | Sex and Age | M 45-64 | 682 | Excess deaths |
|  |  | Sex and Age | M 65 - 74 | 1100 | Excess deaths |
|  |  | Sex and Age | M >=75 | 595 | Excess deaths |
|  |  | Sex and Age | F <15 |  | Excess deaths |
|  |  | Sex and Age | F 15-24 |  | Excess deaths |
|  |  | Sex and Age | F 25-44 | 22 | Excess deaths |
|  |  | Sex and Age | F 45-64 | 166 | Excess deaths |
|  |  | Sex and Age | F 65 - 74 | 487 | Excess deaths |
|  |  | Sex and Age | F >=75 | -729 | Excess deaths |
| Excess natural-cause deaths in California by cause and setting: March 2020 through February 2021 | State, USA | Care setting | In hospital | −626 (−1,324 to 72) | Excess deaths (95%CI) |
|  |  | Care setting | Out of hospital | −43 (−233 to 146) | Excess deaths (95%CI) |

**Table S27. Quality assessment (Newcastle Ottawa Scale).**

| **Study title** | **Selection** | | | | **Comparability** | **Outcome** |  |  | **Score** | **Evaluation** |
| --- | --- | --- | --- | --- | --- | --- | --- | --- | --- | --- |
|  | **Representativeness of the exposed cohort** | **Selection of the non exposed cohort** | **Ascertainment of exposure** | **Demonstration that outcome of interest was not present at start of study** | **Comparability of cohorts on the basis of the design or analysis** | **Assessment of outcome** | **Was follow-up long enough for outcomes to occur?** | **Adequacy of follow up of cohorts** |  |  |
| Excess of cardiovascular deaths during the COVID-19 pandemic in Brazilian capital cities | a) truly representative of the average cardiovascular deaths in the community * | a) drawn from the same community as the exposed cohort * | a) secure record (eg surgical records) * | a) yes * |  | b) record linkage * | a) yes (select an adequate follow up period for outcome of interest) * | a) complete follow up - all subjects accounted for * | 7 | fair |
| Excess mortality by specific causes of deaths in the city of São Paulo, Brazil, during the COVID-19 pandemic | a) truly representative of the mortality by specific causes of deaths in the community * | a) drawn from the same community as the exposed cohort * | a) secure record (eg surgical records) * | a) yes * | a) study controls for sex and age * | b) record linkage * | a) yes (select an adequate follow up period for outcome of interest) * | a) complete follow up - all subjects accounted for * | 8 | good |
| Counting the Dead: COVID-19 and Mortality in Quebec and British Columbia During the First Wave | a) truly representative of the mortality in the community * | a) drawn from the same community as the exposed cohort * | a) secure record (eg surgical records) * | a) yes * |  | b) record linkage * | a) yes (select an adequate follow up period for outcome of interest) * | a) complete follow up - all subjects accounted for * | 7 | fair |
| Excess mortality in Wuhan city and other parts of China during the three months of the covid-19 outbreak: findings from nationwide mortality registries | a) truly representative of the cause specific mortality in the community * | a) drawn from the same community as the exposed cohort * | a) secure record (eg surgical records) * | a) yes * |  | b) record linkage * | a) yes (select an adequate follow up period for outcome of interest) * | a) complete follow up - all subjects accounted for * | 7 | fair |
| Temporal dynamic in the impact of COVID- 19 outbreak on cause-specific mortality in Guangzhou, China | a) truly representative of the cause-specific mortality in the community * | a) drawn from the same community as the exposed cohort * | a) secure record (eg surgical records) * | a) yes * |  | b) record linkage * | a) yes (select an adequate follow up period for outcome of interest) * | a) complete follow up - all subjects accounted for * | 7 | fair |
| Substantial decline in hospital admissions for heart failure accompanied by increased community mortality during COVID-19 pandemic | a) truly representative of the average mortality for heart failure in the community * | a) drawn from the same community as the exposed cohort * | a) secure record (eg surgical records) * | a) yes * |  | b) record linkage * | a) yes (select an adequate follow up period for outcome of interest) * | a) complete follow up - all subjects accounted for * | 7 | fair |
| Place and Underlying Cause of Death During the COVID-19 Pandemic: Retrospective Cohort Study of 3.5 Million Deaths in England and Wales, 2014 to 2020 | a) truly representative of the cause of death in the community * | a) drawn from the same community as the exposed cohort * | a) secure record (eg surgical records) * | a) yes * |  | b) record linkage * | a) yes (select an adequate follow up period for outcome of interest) * | a) complete follow up - all subjects accounted for * | 7 | fair |
| Effects of COVID-19 on mortality: A 5-year population-based study in Oman | a) truly representative of the mortality rate in the community * | a) drawn from the same community as the exposed cohort * | a) secure record (eg surgical records) * | a) yes * |  | b) record linkage * | a) yes (select an adequate follow up period for outcome of interest) * | a) complete follow up - all subjects accounted for * | 7 | fair |
| Cardiovascular-related deaths at the beginning of the COVID-19 outbreak: a prospective analysis based on the UK Biobank | b) somewhat representative of the population with cardiovascular disease (UK Biobank) in the community * | a) drawn from the same community as the exposed cohort * | a) secure record (eg surgical records) * | a) yes * | a) study controls for seasonal variations and age * | b) record linkage * | a) yes (select an adequate follow up period for outcome of interest) * | a) complete follow up - all subjects accounted for * | 8 | good |
| Place and causes of acute cardiovascular mortality during the COVID-19 pandemic | a) truly representative of the average CV events in the community (Civil Registration Deaths Data of the Office for National Statistics of England and Wales) * | a) drawn from the same community as the exposed cohort * | a) secure record (eg surgical records) * | a) yes * | a) study controls for seasonality * | b) record linkage * | a) yes (select an adequate follow up period for outcome of interest) * | a) complete follow up - all subjects accounted for * | 8 | good |
| Excess deaths from COVID-19 and other causes by region, neighbourhood deprivation level and place of death during the first 30 weeks of the pandemic in England and Wales: A retrospective registry study | a) truly representative of the average mortality rate in the community * | a) drawn from the same community as the exposed cohort * | a) secure record (eg surgical records) * | a) yes * |  | b) record linkage * | a) yes (select an adequate follow up period for outcome of interest) * | a) complete follow up - all subjects accounted for * | 7 | fair |
| Excess Cerebrovascular Mortality in the United States During the COVID-19 Pandemic | a) truly representative of the average excess cerebrovascular deaths in the community * | a) drawn from the same community as the exposed cohort * | a) secure record (eg surgical records) * | a) yes * |  | b) record linkage * | a) yes (select an adequate follow up period for outcome of interest) * | a) complete follow up - all subjects accounted for * | 7 | fair |
| Mortality From Drug Overdoses, Homicides, Unintentional Injuries, Motor Vehicle Crashes, and Suicides During the Pandemic, March-August 2020 | a) truly representative of the average cause-specific mortality in the community * | a) drawn from the same community as the exposed cohort * | a) secure record (eg surgical records) * | a) yes * |  | b) record linkage * | a) yes (select an adequate follow up period for outcome of interest) * | a) complete follow up - all subjects accounted for * | 7 | fair |
| Variation in Cause-Specific Mortality Rates in Italy during the First Wave of the COVID-19 Pandemic: A Study Based on Nationwide Data | a) truly representative of the average cause specific mortality in the community * | a) drawn from the same community as the exposed cohort * | a) secure record (eg surgical records) * | a) yes * | a) study controls for sex * | b) record linkage * | a) yes (select an adequate follow up period for outcome of interest) * | a) complete follow up - all subjects accounted for * | 8 | good |
| Leading causes of excess mortality in Mexico during the COVID-19 pandemic 2020-2021: A death certificates study in a middle-income country | a) truly representative of the average leading causes of mortality in the community * | a) drawn from the same community as the exposed cohort * | a) secure record (eg surgical records) * | a) yes * |  | b) record linkage * | a) yes (select an adequate follow up period for outcome of interest) * | a) complete follow up - all subjects accounted for * | 7 | fair |
| Excess mortality associated with the COVID-19 pandemic in Latvia: a population-level analysis of all-cause and noncommunicable disease deaths in 2020 | a) truly representative of the average all-cause and cause-specific weekly mortality in the community * | a) drawn from the same community as the exposed cohort * | a) secure record (eg surgical records) * | a) yes * |  | b) record linkage * | a) yes (select an adequate follow up period for outcome of interest) * | a) complete follow up - all subjects accounted for * | 7 | fair |
| Impact of the COVID-19 Pandemic on Total and Cause-Specific Mortality in Pavia, Northern Italy | a) truly representative of the overall and cause-specific mortality in the community * | a) drawn from the same community as the exposed cohort * | a) secure record (eg surgical records) * | a) yes * | a) study controls for sex and age * | b) record linkage * | a) yes (select an adequate follow up period for outcome of interest) * | a) complete follow up - all subjects accounted for * | 8 | good |
| Covid-19 in Brazil in 2020: impact on deaths from cancer and cardiovascular diseases | a) truly representative of the mortality from cancer and cardiovascular diseases in the community * | a) drawn from the same community as the exposed cohort * | a) secure record (eg surgical records) * | a) yes * | a) study controls for age and territory * | b) record linkage * | a) yes (select an adequate follow up period for outcome of interest) * | a) complete follow up - all subjects accounted for * | 8 | good |
| The US Midlife Mortality Crisis Continues: Excess Cause-Specific Mortality During 2020 | a) truly representative of the monthly mortality in the community * | a) drawn from the same community as the exposed cohort * | a) secure record (eg surgical records) * | a) yes * | a) study controls for sex and age * | b) record linkage * | a) yes (select an adequate follow up period for outcome of interest) * | a) complete follow up - all subjects accounted for * | 8 | good |
| Excess suicides in Brazil: Inequalities according to age groups and regions during the COVID-19 pandemic | a) truly representative of the mortality for suicide in the community * | a) drawn from the same community as the exposed cohort * | a) secure record (eg surgical records) * | a) yes * |  | b) record linkage * | a) yes (select an adequate follow up period for outcome of interest) * | a) complete follow up - all subjects accounted for * | 7 | fair |
| Child mortality in England during the first year of the COVID-19 pandemic | a) truly representative of the average child mortality in the community * | a) drawn from the same community as the exposed cohort * | a) secure record (eg surgical records) * | a) yes * |  | b) record linkage * | a) yes (select an adequate follow up period for outcome of interest) * | a) complete follow up - all subjects accounted for * | 7 | fair |
| Excess natural-cause deaths in California by cause and setting: March 2020 through February 2021 | a) truly representative of the mortality in the community * | a) drawn from the same community as the exposed cohort * | a) secure record (eg surgical records) * | a) yes * |  | b) record linkage * | a) yes (select an adequate follow up period for outcome of interest) * | a) complete follow up - all subjects accounted for * | 7 | fair |
